# Signal Peptidase-Mediated Cleavage of the Anti-σ Factor RsiP at Site 1 Controls σ^P^ Activation and β-Lactam Resistance in Bacillus thuringiensis

**DOI:** 10.1128/mbio.03707-21

**Published:** 2022-02-15

**Authors:** Kelsie M. Nauta, Theresa D. Ho, Craig D. Ellermeier

**Affiliations:** a Department of Microbiology and Immunology, Carver College of Medicine, University of Iowa, Iowa City, Iowa, USA; b Graduate Program in Genetics, University of Iowa, Iowa City, Iowa, USA; Yale School of Medicine

**Keywords:** σ factors, cell envelope, stress response, signal transduction, gene expression, sigma factors

## Abstract

In Bacillus thuringiensis, β-lactam antibiotic resistance is controlled by the extracytoplasmic function (ECF) σ factor σ^P^. σ^P^ activity is inhibited by the anti-σ factor RsiP. In the presence of β-lactam antibiotics, RsiP is degraded and σ^P^ is activated. Previous work found that RsiP degradation requires cleavage of RsiP at site 1 by an unknown protease, followed by cleavage at site 2 by the site 2 protease RasP. The penicillin-binding protein PbpP acts as a sensor for β-lactams. PbpP initiates σ^P^ activation and is required for site 1 cleavage of RsiP but is not the site 1 protease. Here, we describe the identification of a signal peptidase, SipP, which cleaves RsiP at a site 1 signal peptidase cleavage site and is required for σ^P^ activation. Finally, many B. anthracis strains are sensitive to β-lactams yet encode the σ^P^-RsiP signal transduction system. We identified a naturally occurring mutation in the signal peptidase cleavage site of B. anthracis RsiP that renders it resistant to SipP cleavage. We find that B. anthracis RsiP is not degraded in the presence of β-lactams. Altering the B. anthracis RsiP site 1 cleavage site by a single residue to resemble B. thuringiensis RsiP results in β-lactam-dependent degradation of RsiP. We show that mutation of the B. thuringiensis RsiP cleavage site to resemble the sequence of B. anthracis RsiP blocks degradation by SipP. The change in the cleavage site likely explains many reasons why B. anthracis strains are sensitive to β-lactams.

## INTRODUCTION

Extracytoplasmic function (ECF) σ factors are specialized σ factors that interact with RNA polymerase to transcribe genes involved in stress responses. ECF σ factors belong to the σ^70^ family of ECF σ factors but lack the σ_3_ and σ_1_ domains (1). More than 150 groups of ECF σ factors have been identified based on sequence homology, but the mechanisms controlling activation of many of these ECF σ factors remain poorly understood (2). Most ECF σ factors are sequestered by anti-σ factors which bind to the σ factor and prevent interaction with RNA polymerase. To activate the σ factor signal transduction cascade, the anti-σ factor must be inactivated to allow the σ factor to interact with RNA polymerase and transcribe target genes. Several mechanisms for anti-σ factor inactivation have been described, allowing further categorization of ECF σ factors (1). One of the more common mechanisms for anti-σ factor inactivation is regulated intramembrane proteolysis (RIP), which results in the proteolytic destruction of anti-σ (3–8). RIP consists of at least two sequential cleavage events, one occurring on the extracellular or periplasmic domain of the anti-σ factor (site 1) and the second occurring within the intramembrane region of the anti-σ factor (site 2) (3–8). Proteolytic cleavage releases the anti-σ–σ factor complex from the membrane. The remainder of the anti-σ factor is destroyed by cytosolic proteases freeing the σ-factor to promote the transcription of target genes.

Although many ECF σ factors are controlled by RIP, there are distinct differences among the most studied systems. The best-studied example is RseA, which inhibits σ^E^ (9). σ^E^ is an ECF σ factor, first described in Escherichia coli, that responds to the presence of misfolded outer membrane (OM) β-barrel proteins in the periplasm (10–12). σ^E^ is released when the cognate anti-σ, RseA, is degraded by sequential cleavage of DegS at site 1 and then of RseP at site 2 (3, 13). The trigger for site 1 cleavage is outer membrane damage. Outer membrane damage can cause the accumulation of unfolded outer membrane β-barrel proteins in the periplasm that bind to a PDZ domain on DegS, allowing it to cleave RseA at site 1 (12). In addition, RseB normally binds to RseA to protect it from cleavage at site 1 (14, 15). However, OM damage leads to periplasmic accumulation of lipopolysaccharide (LPS). The binding of LPS to RseB releases RseB from RseA so that RseA can be recognized by DegS (14–17). Following site 2 cleavage, the cytoplasmic portion of RseA is primarily degraded by ClpPX. Degradation of RseA frees σ^E^ to interact with RNA polymerase and to transcribe target genes involved in outer membrane biogenesis (15).

σ^W^ is a second example of an ECF σ factor that is activated by RIP. σ^W^ is present in Bacillus subtilis and is involved in the response to antimicrobial peptides (5, 18, 19). The activity of σ^W^ is inhibited by the anti-σ factor RsiW. RsiW is degraded following exposure to antimicrobial peptides. RsiW is first cleaved by PrsW at site 1 (5, 20). Then an unidentified protease further trims RsiW before RasP, a homologue of RseP, can cleave RsiW within the transmembrane domain at site 2 (4, 21). The cytoplasmic portion of RsiW is degraded by Clp proteases (22). σ^W^ then induces the expression of genes involved in cell envelope biogenesis and resistance to antimicrobial peptides (5, 19).

More recent work has shown that the ECF σ factor σ^V^ is activated by RIP. σ^V^ is encoded in B. subtilis, Clostridioides difficile, and Enterococcus faecalis and induces resistance to lysozyme (23–27). The activity of σ^V^ is inhibited by the anti-σ factor RsiV (28, 29). σ^V^ is activated when RsiV binds lysozyme (30, 31). RsiV binding to lysozyme allows RsiV to be cleaved at site 1 by the type I signal peptidase SipS or SipT, followed by cleavage at site 2 by RasP (6, 30). Presumedly, the cytoplasmic portion of RsiV is degraded by cytosolic proteases. σ^V^ then induces the expression of lysozyme resistance genes (25, 32–35). RsiV is not cleaved in the absence of lysozyme because the signal peptidase cleavage site is embedded within an amphipathic helix (36). This helix becomes exposed when lysozyme binds RsiV, allowing SipS or SipT to cleave at site 1 (36).

Type I signal peptidases, such as B. subtilis SipT, SipS, SipU, and SipV, are membrane-bound serine proteases that cleave the leader peptide of secreted proteins. Signal peptide cleavage frees the proteins from the membrane and enables secretion or localization to the periplasm or outer membrane (37–39). The residues at amino acid positions −3 and −1 with respect to the signal peptidase cleavage site are typically small, noncharged amino acids (38, 40). These residues are often alanine, making the most common signal peptidase cleavage site AXA (40). SipS and SipT are the major signal peptidases in B. subtilis and are redundant, as *sipS* and *sipT* can be deleted individually; however, deletion of both is lethal (39, 41). The transcription of the signal peptidases SipS and SipT is temporally regulated, with maximum expression occurring after exponential phase. SipU and SipV are constitutively expressed at low levels (39, 42).

In Bacillus thuringiensis and Bacillus cereus, the ECF σ factor σ^P^ is sequestered by the anti-σ factor RsiP, a single-pass transmembrane protein (43). In the presence of ampicillin, methicillin, cefoxitin, cephalothin, cefmetazole, and cefalexin, RsiP is degraded and σ^P^ is released to upregulate the expression of β-lactamases (8). The β-lactams piperacillin, cefsulodin, and cefoperazone do not activate σ^P^ (8). RsiP degradation is a multistep process that requires cleavage in the extracellular domain of RsiP by an unknown protease at site 1 (8). Site 1 cleavage is quickly followed by site 2 cleavage within the transmembrane domain of RsiP by the site 2 protease RasP (8). Previous work indicates that site 1 cleavage is the rate-limiting step in σ^P^ activation (8). While the site 1 protease is unknown, the penicillin-binding protein PbpP was found to be required for σ^P^ activation and RsiP degradation (44). Importantly, PbpP likely does not cleave RsiP at site 1 but is required to induce site 1 cleavage likely via a protein-protein interaction (44). We hypothesize that PbpP acts as a sensor for β-lactams and initiates the signaling cascade resulting in degradation of RsiV and activation of σ^P^.

We sought to identify the site 1 protease that cleaves RsiP in response to σ^P^-inducing β-lactams to better inform our understanding of how σ^P^ is activated. We identified a predicted noncanonical signal peptidase cleavage site in RsiP and showed that mutation of the cleavage site prevents RsiP cleavage at site 1. We also demonstrated that the predicted signal peptidase, SipP (HD73_4122), is necessary and sufficient for cleavage of RsiP at site 1. Overexpression of *sipP* activates σ^P^, but activation is reduced in the absence of *pbpP*. Using B. subtilis (which does not encode σ^P^-RsiP), we reconstituted the known components required for σ^P^ activation. We showed that σ^P^ activity is controlled in a β-lactam-dependent manner. This suggests that if there is an unknown component, it is conserved between B. thuringiensis and B. subtilis.

While Bacillus anthracis encodes *sigP* and *rsiP*, σ^P^ is not activated in response to ampicillin in most strains (43). Many B. anthracis strains are sensitive to β-lactams, and σ^P^ is not activated by β-lactams (43, 45, 46). However, the β-lactam-resistant strains of B. anthracis carry truncated versions of *rsiP* that lack the σ^P^ binding domain and constitutively express σ^P^ (43, 47, 48). Ross and colleagues showed that complementation of a B. anthracis *sigP-rsiP* mutant with *sigP-rsiP* from B. cereus or B. thuringiensis resulted in inducible σ^P^ activity (43). Thus, RsiP from B. anthracis likely lacks the ability to respond to ampicillin (43). However, the difference between B. anthracis *rsiP* and B. thuringiensis *rsiP* is not known. We provide evidence that a point mutation in the signal peptidase cleavage site of RsiP from Bacillus anthracis prevents cleavage at site 1. We show that reversal of this point mutation to the B. thuringiensis sequence restores RsiP degradation in the presence of β-lactams.

## RESULTS

### Identification of a putative signal peptidase cleavage site in RsiP.

**FIG 1:**
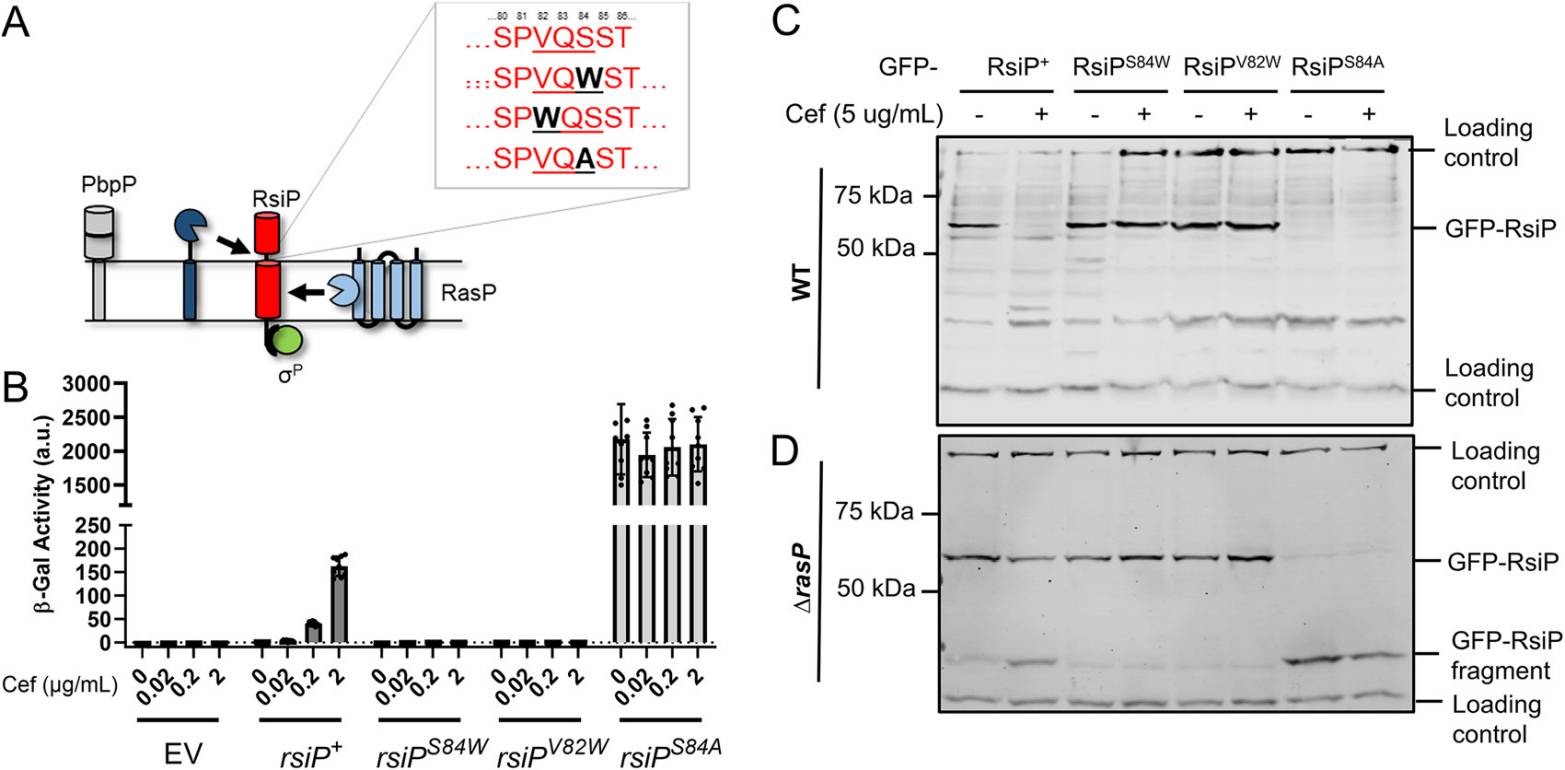
A predicted signal peptidase cleavage site is required for RsiP degradation. (A) Model. RsiP (red) is cleaved by RasP at site 2 (light blue), but the site 1 protease (dark blue) is unknown. PbpP was shown to play a role in β-lactam sensing and signaling for σ^P^ activation (44). The SignalP (50) predicted signal peptidase cleavage site (VQS) is shown along with the corresponding mutations. (B) Mutation of RsiP results in a loss of σ^P^ activation. All strains contain the reporter *P_sigP_-lacZ* integrated into the *thrC* locus. The relevant genotypes are WT (THE2549), *rsiP* (EBT238), *rsiP^S84W^* (EBT1136), *rsiP^V82W^* (EBT1165), and *rsiP^S84A^* (EBT1166). Cells were grown to mid-log phase (OD_600_, 0.6 to 0.8) at 37°C. Cefoxitin (0.02 to 2 μg/mL) was added, and the cells were incubated for 1 h at 37°C. β-Galactosidase activities were calculated as described in Materials and Methods. Experiments were performed in technical and biological triplicates, and standard deviations are represented by error bars. a.u., arbitrary units. (C, D) Mutation of the predicted signal peptidase cleavage site inhibits RsiP degradation. A GFP-RsiP fusion was used with the following RsiP mutants in WT RsiP^+^ (EBT936), RsiP^S84W^ (EBT1207), RsiP^V82W^ (EBT1209), and RsiP^S84A^ (EBT1208) (C) or in *ΔrasP* mutant Δ*rasP*/RsiP^+^ (EBT939), Δ*rasP*/RsiP^S84W^ (EBT1210), Δ*rasP*/RsiP^V82W^ (EBT1212), and Δ*rasP*/RsiP^S84A^ (EBT1211) (D) strains. Cells were grown to mid-log phase (OD_600_, 0.6 to 0.8) with 1 mM IPTG at 37°C, pelleted, and concentrated in LB or LB plus cefoxitin (5 μg/mL). Cells were incubated for 1 h at 37°C before sample buffer was added. Immunoblotting was performed using anti-GFP antisera. Streptavidin IR680LT was used to detect PycA (HD73_4231) and AccB (HD73_4487), which served as a loading control (68, 69). The color blot with both anti-GFP and streptavidin on a single gel is shown in Fig. S2A and B in the supplemental material. Numbers at the left indicate the molecular masses (in kilodaltons) of the ladder. The bands corresponding to the loading control and GFP-RsiP fragments are indicated on the right.

Previous work demonstrated that RsiP is cleaved at site 2 by RasP in the presence of cefoxitin (8). We recently demonstrated that the penicillin-binding protein PbpP is required for site 1 cleavage of RsiP but is likely not the site 1 protease (44). Thus, we sought to identify the protease that cleaves RsiP at site 1. In B. subtilis, σ^V^ is activated when RsiV binds to lysozyme, which triggers the cleavage of RsiV at site 1 by the signal peptidases SipS and SipT (6, 36). The RsiV signal peptidase cleavage site lies within an amphipathic helix and is part of a domain of unknown function, DUF4179 (36). RsiP does not contain the DUF4179 domain or an obvious amphipathic helix; however, we noted that site 1 cleavage of RsiP is reduced in *ΔrasP* cells (8). In B. subtilis, signal peptidase activity is reduced in *rasP* mutants (49). Using the SignalP 5.0 software (50), we identified a weak potential signal peptidase cleavage site, VQS, in RsiP near the transmembrane domain in the extracellular portion of RsiP (see Fig. S1A in the supplemental material). We found that *in silico* mutation of either V82 or S84 to a tryptophan abolished the predicted cleavage site. We also found that *in silico* mutation of S84 to an alanine increased the probability of signal peptidase cleavage substantially (Fig. 1A and Fig. S1A).

10.1128/mbio.03707-21.3 FIG S1 SignalP predicts a weak signal peptidase cleavage site in RsiP. The amino acid sequences for WT RsiP^71–276^ as well as for RsiP^S84W^, RsiP^V82W^, and RsiP^S84A^ were submitted to SignalP-5.0 with the following selected setting: Gram positive, long output (50). The data were reconstructed in GraphPad Prism 9.0.0. Download FIG S1, TIF file, 1.6 MB. Copyright © 2022 Nauta et al. 2022 Nauta et al. https://creativecommons.org/licenses/by/4.0/ This content is distributed under the terms of the Creative Commons Attribution 4.0 International license.

10.1128/mbio.03707-21.4 FIG S2 Colored version of Fig. 1C. Mutation of the predicted signal peptidase cleavage site inhibits RsiP degradation. The relevant genotypes are RsiP^+^ (EBT936), RsiP^S84W^ (EBT1207), RsiP^V82W^ (EBT1209), and RsiP^S84A^ (EBT1208) (top) and Δ*rasP*/RsiP^+^ (EBT939), Δ*rasP*/RsiP^S84W^ (EBT1210), Δ*rasP*/RsiP^V82W^ (EBT1212), and Δ*rasP*/RsiP^S84A^ (EBT1211) (bottom). Cells were grown to mid-log phase (OD_600_, 0.6 to 0.8) with 1 mM IPTG at 37°C, pelleted, and concentrated in LB or LB plus cefoxitin (5 μg/mL). Cells were incubated for 1 h at 37°C before the sample buffer was added. Immunoblotting was performed using anti-GFP antisera. Streptavidin IR680LT was used to detect PycA (HD73_4231) and AccB (HD73_4487), which served as loading controls (68, 69). Numbers at the left indicate the molecular masses (in kilodaltons) of the ladder. The bands corresponding to the loading control and GFP-RsiP fragments are indicated on the right. Download FIG S2, TIF file, 2.6 MB. Copyright © 2022 Nauta et al. 2022 Nauta et al. https://creativecommons.org/licenses/by/4.0/ This content is distributed under the terms of the Creative Commons Attribution 4.0 International license.

To test these predictions, we examined the effect of these mutations on σ^P^ activation *in vivo* (Fig. 1B). Since *P_sigP_-lacZ* expression is dependent upon σ^P^ activity, we used a derivative of B. thuringiensis serovar kurstaki HD73 that contained a *P_sigP_-lacZ* reporter inserted at the *thrC* locus to monitor σ^P^ activity. In this strain, we deleted the chromosomal copy of *sigP-rsiP.* Into the resulting *sigP-rsiP* deletion report strain, we introduced plasmids carrying either wild-type (WT) *sigP^+^-rsiP^+^*, *sigP^+^-rsiP^S84W^*, *sigP^+^-rsiP^V82W^*, or *sigP^+^-rsiP^S84A^*. We measured β-galactosidase activity using cefoxitin as a model β-lactam because it is a good σ^P^ inducer, and the fold change in MIC between the WT and *ΔsigP-rsiP* mutant, which lacks both SigP and RsiP, is relatively small, compared to that for other inducers (8). As expected, strains with WT *sigP^+^-rsiP^+^* were induced in response to cefoxitin (Fig. 1B). However, the strains carrying the *sigP^+^-rsiP^S84W^* or *sigP^+^-rsiP^V82W^* mutant alleles showed no *P_sigP_-lacZ* expression when grown in the presence of cefoxitin (Fig. 1B). This suggests that these mutations block σ^P^ activation. We also found that *rsiP^S84A^* resulted in high levels of *P_sigP_-lacZ* expression, even in the absence of cefoxitin, suggesting constitutive σ^P^ activation (Fig. 1B).

To determine if these mutations affected RsiP degradation, we constructed green fluorescent protein (GFP) translational fusions to each of the RsiP mutants. We grew cells to mid-log phase and then split the culture and added cefoxitin to one set. We then monitored GFP-RsiP degradation by immunoblotting. In WT cells, we observed the loss of WT GFP-RsiP when cefoxitin was added (Fig. 1C). We found that GFP-RsiP^S84W^ and GFP-RsiP^V82W^ were not degraded in response to cefoxitin (Fig. 1C). This result suggests that cefoxitin-induced cleavage of RsiP at site 1 is blocked in these mutants. Interestingly, we do not detect GFP-RsiP^S84A^, suggesting that it was constitutively degraded (Fig. 1C). Since the absence of the site 2 protease RasP results in the accumulation of the site 1 cleavage product (30-kDa GFP-RsiP fragment), we introduced these fusions into *ΔrasP* mutants. In response to cefoxitin, we found an accumulation of the 30-kDa GFP-RsiP fragment in cells producing GFP-RsiP^+^, but we did not see this accumulation in cells producing GFP-RsiP^S84W^ or GFP-RsiP^V82W^ (Fig. 1D). This suggests that cefoxitin-induced cleavage of RsiP at site 1 is blocked in these mutants. Furthermore, we observed an accumulation of the GFP-RsiP fragment in cells producing GFP-RsiP^S84A^ even in the absence of cefoxitin (Fig. 1D). This suggests that GFP-RsiP^S84A^ is produced but rapidly cleaved at site 1, even in the absence of β-lactam antibiotics. These findings are consistent with the hypothesis that VXS is the site 1 cleavage site of RsiP and suggest that RsiP may be cleaved by signal peptidases.

### SipX and SipP cleave RsiP in Bacillus subtilis.

**FIG 2:**
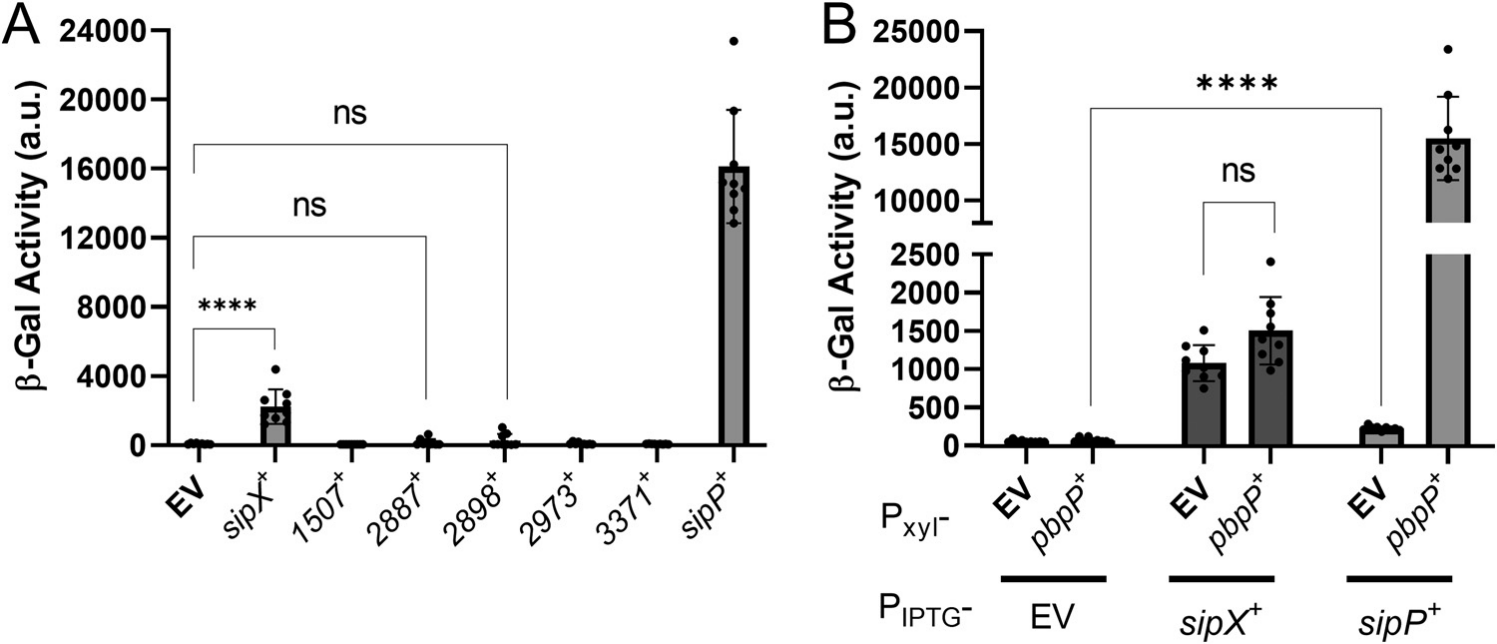
Identification of signal peptidases that are sufficient to activate σ^P^. (A) Expression of B. thuringiensis signal peptidases in B. subtilis. All strains are B. subtilis and contain *P_sigP_-sigP^+^-rsiP^+^-lacZ* and *P_xyl_-pbpP^+^*. The strain-specific relevant genotypes are the empty vector (EV) (CDE3602), *P*_IPTG_*-bt2887* (CDE3603), *P*_IPTG_-*sipX* (CDE3604), *P*_IPTG_*-bt1507* (CDE3605), *P*_IPTG_*-bt2973* (CDE3606), *P*_IPTG_*-bt3371* (CDE3608), *P*_IPTG_-*sipP* (CDE3610), and *P*_IPTG_*-bt2898* (CDE3612). The strains were grown in the presence of xylose (1%) and IPTG (1 mM) at 37°C to an OD_600_ of 1.6 to 1.8. β-Galactosidase activities were calculated as described in Materials and Methods. Experiments were performed in technical and biological triplicates, and standard deviations are represented by error bars. ns, not significant. (B) All strains are B. subtilis and contain *P_sigP_-sigP-rsiP-lacZ* and the following relevant genotypes: *P*_IPTG_ (CDE3613), *P*_IPTG_ *P_xyl_-pbpP* (CDE3602), *P*_IPTG_*-sipX* (CDE3614), *P*_IPTG_*-sipX P_xyl_-pbpP* (CDE3604), *P*_IPTG_*-sipP* (CDE3615), and *P*_IPTG_*-sipP P_xyl_-pbpP* (CDE3610). Cells were prepared and β-galactosidase activities were determined using the same methods as described for panel A. Strains grown in the absence of IPTG are shown in Fig. S3A and S3B. ****, *P* value of <0.0001.

Our data suggest that RsiP is cleaved at a putative signal peptidase cleavage site. We previously showed that overexpression of the penicillin-binding protein PbpP induces site 1 cleavage of RsiP in B. thuringiensis but not in B. subtilis (44). This suggests that B. subtilis lacks the site 1 protease. Thus, we used B. subtilis as a heterologous host to identify a site 1 protease which could cleave RsiP. We constructed a B. subtilis strain that contains *P_sigP_-sigP-rsiP-lacZ* and *P_xyl_-pbpP* and utilized endogenous B. subtilis RasP for site 2 protease activity. We found that production of PbpP does not induce *P_sigP_-lacZ* expression (Fig. 2A). This result is consistent with our previous observation that production of PbpP does not induce RsiP degradation in B. subtilis (44). Thus, if a signal peptidase is involved in site 1 cleavage of RsiP, it must be a B. thuringiensis-specific signal peptidase.

10.1128/mbio.03707-21.5 FIG S3 Induction differences with and without IPTG (refer to Fig. 2). Identification of signal peptidases that are sufficient to activate σ^P^. (A) Expression of B. thuringiensis signal peptidases in B. subtilis. All strains contain *P_sigP_-sigP^+^-rsiP^+^-lacZ* and *P_xyl_-pbpP^+^*. The strain-specific relevant genotypes are EV (CDE3602), *P*_IPTG_*-bt2887* (CDE3603), *P*_IPTG_*-sipX* (CDE3604), *P*_IPTG_*-bt1507* (CDE3605), *P*_IPTG_-*bt2973* (CDE3606), *P*_IPTG_-*bt3371* (CDE3608), *P*_IPTG_-*sipP* (CDE3610), and *P*_IPTG_*-bt2898* (CDE3612). The strains were grown in the presence or absence of xylose (1%) and IPTG (1 mM) at 37°C to an OD_600_ of 1.6 to 1.8. β-Galactosidase activities were calculated as described in Materials and Methods. Experiments were performed in technical and biological triplicates, and standard deviations are represented by error bars. (B) All strains contain *P_sigP_-sigP-rsiP-lacZ* and the following relevant genotypes: *P*_IPTG_ (CDE3613), *P*_IPTG_ *P_xyl_-pbpP* (CDE3602), *P*_IPTG_*-sipX* (CDE3614), *P*_IPTG_*-sipX P_xyl_-pbpP* (CDE3604), *P*_IPTG_*-sipP* (CDE3615), and *P*_IPTG_*-sipP P_xyl_-pbpP* (CDE3610). Cells were prepared and β-galactosidase activities were determined using the same methods as described for Fig. 2A. Download FIG S3, TIF file, 1.3 MB. Copyright © 2022 Nauta et al. 2022 Nauta et al. https://creativecommons.org/licenses/by/4.0/ This content is distributed under the terms of the Creative Commons Attribution 4.0 International license.

We identified seven predicted type I signal peptidases in B. thuringiensis HD73 using BLASTP (51). To identify which of the signal peptidases cleave RsiP at site 1, we cloned each of them under the control of an isopropyl-β-d-thiogalactopyranoside (IPTG)-inducible promoter (*P*_IPTG_) and integrated the constructs at the ICE*Bs1* site of the B. subtilis chromosome in a strain that contained *P_sigP_-sigP-rsiP-lacZ* and *P_xyl_-pbpP.* As noted, expression of PbpP alone does not induce *P_sigP_-lacZ* expression (Fig. 2A). We found that expression of PbpP plus either HD73_0543 or HD73_4122 (here referred to as SipX and SipP, respectively) induced *P_sigP_-lacZ* expression (Fig. 2A). This result suggests that SipX and SipP are likely able to cleave RsiP at site 1, which leads to σ^P^ activation.

As noted in B. thuringiensis, PbpP is required for activation of σ^P^ in response to β-lactams (44). Thus, we tested if SipX and SipP could induce *P_sigP_-lacZ* expression in the absence of PbpP in B. subtilis. We found that when *sipP* was expressed in the absence of PbpP, it failed to induce *P_sigP_-lacZ* expression, suggesting that σ^P^ activation was dependent on the presence of PbpP (Fig. 2B). In contrast, we found that activation of σ^P^ by SipX was not dependent on PbpP (Fig. 2B). These data suggest that while either SipX or SipP is sufficient for cleavage of RsiP at site 1, only SipP cleavage requires PbpP. This suggests that SipP is likely the site 1 protease and that overexpression of SipX likely forces artificial cleavage of RsiP.

### SipP is required for σ^P^ activation in B. thuringiensis.

**FIG 3:**
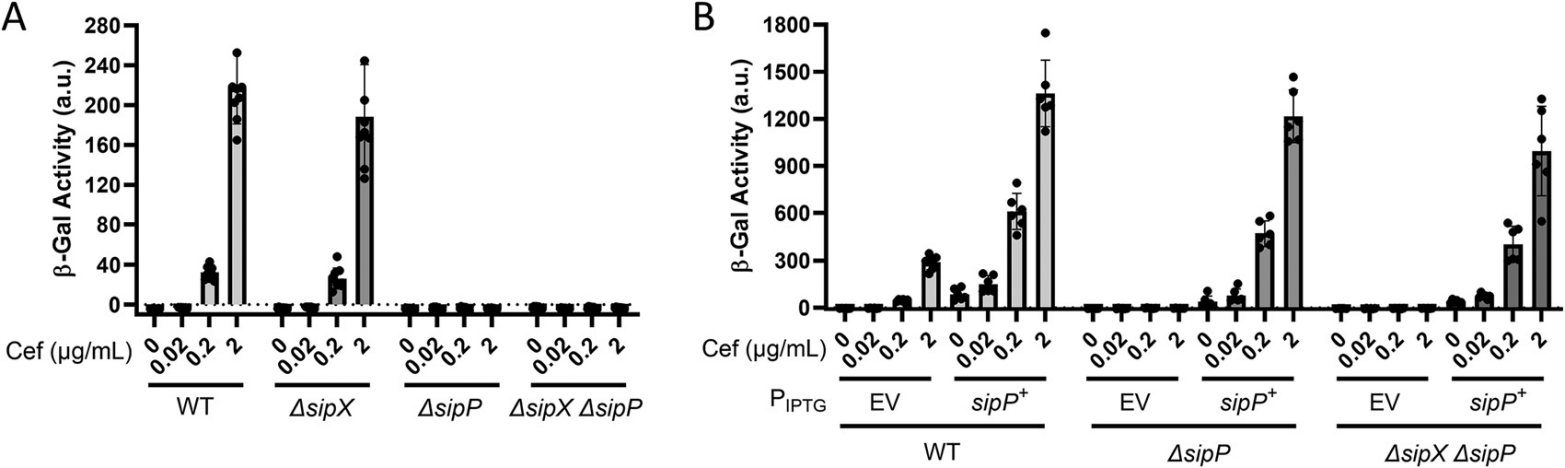
SipP is required for σ^P^ activation in B. thuringiensis. (A) SipP is required for σ^P^ activation. All strains contain *P_sigP_-lacZ* and the following relevant genotypes: WT (THE2549), *ΔsipX* (EBT1170), Δ*sipP* (EBT1202), and *ΔsipX* Δ*sipP* (EBT1213). (B) *sipP* complements *ΔsipP.* All strains contain *P_sigP_-lacZ* and the following relevant genotypes: WT/EV (EBT728), WT/*P*_IPTG_*-sipP* (EBT1269), *ΔsipP*/EV (EBT1244), *ΔsipP/P*_IPTG_*-sipP* (EBT1218), *ΔsipX ΔsipP/*EV (EBT1246), and *ΔsipX ΔsipP/P*_IPTG_*-sipP* (EBT1220). All strains were grown to mid-log phase (OD_600_, 0.6 to 0.8) at 37°C. Cefoxitin (Cef, 0.02 to 2 μg/mL) was added, and the cells were incubated for another hour at 37°C. Cells were prepared and β-galactosidase activities were determined using the same methods as described for panel A. Experiments were performed in technical and biological triplicates, and standard deviations are represented by error bars.

To determine if SipX and SipP are required for σ^P^ activation, we constructed in-frame deletions of each gene in B. thuringiensis HD73 *P_sigP_-lacZ* (WT), generating *ΔsipX* and *ΔsipP* strains. We also constructed a *ΔsipX ΔsipP* double mutant. We incubated these strains in the presence of increasing concentrations of cefoxitin and found that expression of *P_sigP_-lacZ* was unaffected by the absence of SipX (Fig. 3A). However, loss of SipP blocked *P_sigP_-lacZ* induction in response to cefoxitin, suggesting that σ^P^ was not activated. We found that the *ΔsipX ΔsipP* double mutant blocked *P_sigP_-lacZ*, as it did with the *ΔsipP* mutant (Fig. 3A). These data suggest that SipP is required for σ^P^ activation but that SipX is not. We hypothesize that artificial overexpression of SipX was sufficient to force the cleavage of RsiP, but under physiological conditions, SipX does not cleave RsiP. We also complemented the *ΔsipP* mutant by expressing *sipP* from an IPTG-inducible promoter and found that *sipP* expression restored σ^P^ activation in response to cefoxitin in both the *ΔsipP* and *ΔsipX ΔsipP* mutants (Fig. 3B). This further suggests that SipP cleaves RsiP at site 1 and that SipX cleavage of RsiP is not physiologically relevant.

As previously reported, the loss of σ^P^ activation results in a loss of β-lactam resistance (8, 43, 44). To determine if a loss of *sipP* resulted in a loss of β-lactam resistance, we determined the MICs of ampicillin, cefoxitin, and cefsulodin for the WT, Δ*sigP-rsiP*, Δ*pbpP*, Δ*sipP*, *ΔsipX*, and *ΔsipX* Δ*sipP* strains. We used cefsulodin as a negative control because σ^P^ does not control resistance to cefsulodin. As with previous reports, we found that the Δ*sigP-rsiP* strain was significantly more sensitive to ampicillin and cefoxitin than the WT (Tables 1 and 2) (8, 44). We also found that the Δ*pbpP* mutant was more sensitive than the WT but less sensitive than the Δ*sigP-rsiP* strain. Both the Δ*sipP* and *ΔsipX* Δ*sipP* mutants were more sensitive to ampicillin and cefoxitin than the WT and Δ*pbpP* mutant but not as sensitive as the Δ*sigP rsiP* mutant (Tables 1 and 2). We hypothesize that the MICs for the Δ*pbpP* and Δ*sipP* mutants are higher than that for the Δ*sigP-rsiP* mutant because these strains have higher basal levels of σ^P^ and therefore higher basal levels of expression of β-lactamases and PBPs. Again, we found that *ΔsipX* had no effect, further reinforcing our hypothesis that it does not play a role in σ^P^ activation.

**TABLE 1:** MICs for different strains

β-Lactam	MIC (μg/mL) (mean ± SD) for:					
	WT	Δ*sigP-rsiP* mutant	Δ*pbpP* mutant	Δ*sipP* mutant	Δ*0543* mutant	Δ*0543* Δ*sipP* mutant
Ampicillin	8,300 ± 3,100	0.104 ± 0.031	1.53 ± 0.773	0.556 ± 0.304	8,300 ± 3,100	0.326 ± 0.207
Cefoxitin	33 ± 12.5	5.2 ± 1.57	6.94 ± 2.08	5.56 ± 1.38	38.9 ± 13.1	4.16 ± 1.57
Cefsulodin	250 ± 75	267 ± 66	300 ± 0	217 ± 79.0	400 ± 150	233 ± 79

**TABLE 2:** Fold differences in MICs

β-Lactam	Fold difference in MICs between:				
	WT and Δ*sigP-rsiP* mutant	WT and Δ*pbpP* mutant	WT and Δ*sipP* mutant	WT and Δ*0543* mutant	WT and Δ*0543* Δ*sipP* mutant
Ampicillin	80,000	5,000	15,000	1	25,500
Cefoxitin	6.3	4.8	5.9	0.85	7.93
Cefsulodin	0.93	0.83	1.15	0.62	1.07

**FIG 4:**
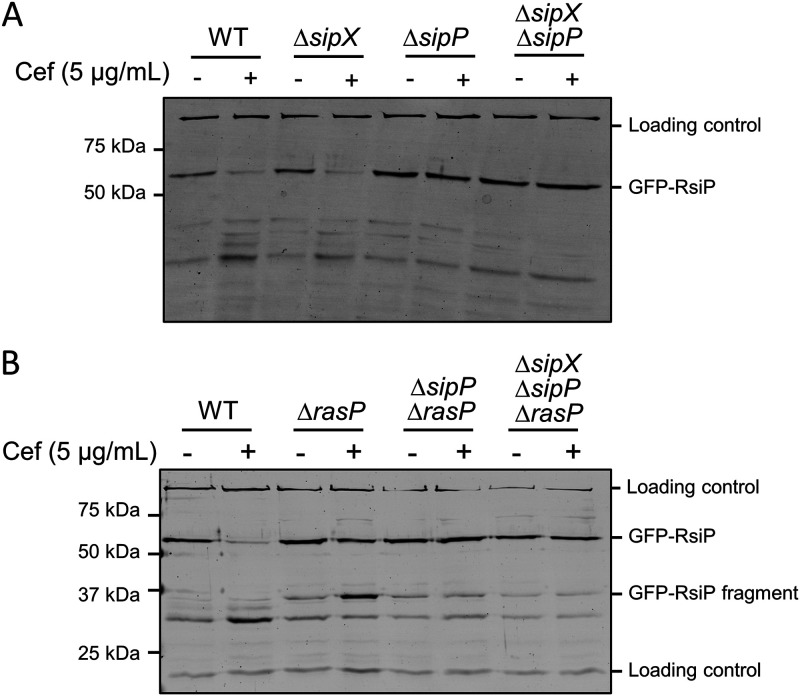
*sipP* is required for degradation of RsiP. All strains contain *P*_IPTG_*-gfp-rsiP^+^* inserted in the ICE*Bs1* locus and the following relevant genotypes: WT (EBT936), *ΔsipX* (EBT1223), *ΔsipP* (EBT1222), and *ΔsipX ΔsipP* (EBT1224) (A) or WT (EBT936), *ΔrasP* (EBT939), *ΔrasP ΔsipP* (EBT1263), and *ΔrasP ΔsipX ΔsipP* (EBT1265) (B). Cells were grown to mid-log phase (OD_600_, 0.6 to 0.8) with 1 mM IPTG at 37°C, pelleted, and concentrated in LB or LB plus cefoxitin (5 μg/mL). Cells were incubated for 1 h at 37°C before sample buffer was added. Immunoblotting was performed using anti-GFP antisera. Streptavidin IR680LT was used to detect PycA (HD73_4231) and AccB (HD73_4487), which served as loading controls (68, 69). The color blot with both anti-GFP and streptavidin on a single gel is shown in Fig. S4A and B. Numbers on the left indicate the molecular masses (in kilodaltons) of the ladder. The bands corresponding to the loading control and GFP-RsiP fragments are indicated on the right.

To determine if SipP was necessary for RsiP degradation, we inserted IPTG-inducible *gfp-rsiP* in the ICE*Bs1* locus in the genome of the WT, *ΔsipX*, *ΔsipP*, and *Δbt0534 ΔsipP* strains. We grew cells in the presence of IPTG and then split and pelleted them, resuspended half of each culture in LB and half in LB with 5 μg/mL cefoxitin, and incubated them for 1 h. We monitored GFP-RsiP degradation by immunoblotting using anti-GFP antibodies. As we previously observed, we found that GFP-RsiP is degraded in the WT in the presence of cefoxitin (8, 44) (Fig. 4A). We also found that the loss of SipX did not alter GFP-RsiP degradation (Fig. 4A). In contrast, we found that GFP-RsiP was not cleaved at site 1 in the Δ*sipP* or *ΔsipX* Δ*sipP* mutant strains (Fig. 4A). This further suggests that SipP is the site 1 protease for RsiP.

10.1128/mbio.03707-21.6 FIG S4 Colored version of Fig. 4. *sipP* is required for degradation of RsiP. All the strains contain *P*_IPTG_*-gfp-rsiP^+^* inserted in the ICE*Bs1* locus and the following relevant genotypes: WT (EBT1130), *ΔsipX* (EBT1223), *ΔsipP* (EBT1222), and *ΔsipX ΔsipP* (EBT1224) (A) or WT (EBT1130), *ΔrasP* (EBT939), *ΔrasP ΔsipP* (EBT1263), and *ΔrasP ΔsipX ΔsipP* (EBT1265) (B). Cells were grown to mid-log phase (OD_600_, 0.6 to 0.8) with 1 mM IPTG at 37°C, pelleted, and concentrated in LB or LB plus cefoxitin (5 μg/mL). Cells were incubated for 1 h at 37°C before sample buffer was added. Immunoblotting was performed using anti-GFP antisera. Streptavidin IR680LT was used to detect PycA (HD73_4231) and AccB (HD73_4487), which served as loading controls (68, 69). Numbers on the left indicate the molecular masses (in kilodaltons) of the ladder. The bands corresponding to the loading control and GFP-RsiP fragments are indicated on the right. Download FIG S4, TIF file, 2.9 MB. Copyright © 2022 Nauta et al. 2022 Nauta et al. https://creativecommons.org/licenses/by/4.0/ This content is distributed under the terms of the Creative Commons Attribution 4.0 International license.

As noted earlier, the site 1 cleavage product (30-kDa GFP-RsiP fragment) accumulates in the absence of RasP. Thus, to determine if site 1 cleavage is blocked in the absence of SipP, we constructed Δ*rasP* Δ*sipP* and Δ*rasP ΔsipX* Δ*sipP* mutants and tracked GFP-RsiP in the presence and absence of cefoxitin in these strains. As previously shown, in the absence of RasP, RsiP is not fully degraded in the presence of cefoxitin (Fig. 4B). We found that the GFP-RsiP fragment did not accumulate in the *ΔrasP ΔsipP* or *ΔrasP ΔsipX ΔsipP* mutant in the presence of cefoxitin (Fig. 4B). All of these data taken together suggest that SipP is required for site 1 cleavage of RsiP in response to cefoxitin.

### SipP is epistatic to PbpP.

**FIG 5:**
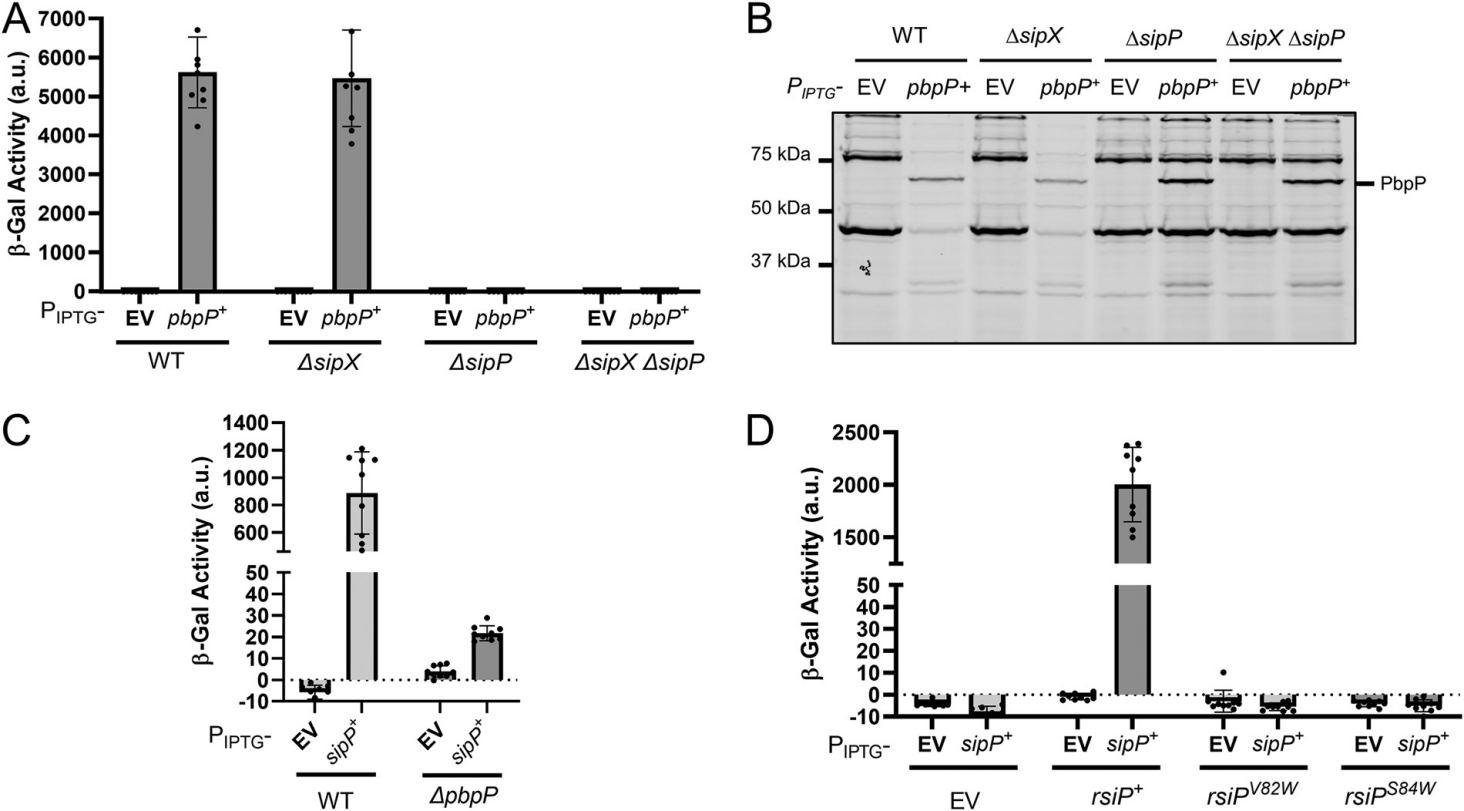
*sipP* is required for activation of σ^P^ by overexpression of *pbpP.* (A) *pbpP* overexpression does not activate σ^P^ in the absence of *sipP*. All strains contain *P_sigP_-lacZ* inserted into the *thrC* locus and the following relevant genotypes: WT/EV (EBT728), WT/*P*_IPTG_*-pbpP^+^* (EBT1239), *ΔsipX*/EV (EBT1242), *ΔsipX*/*P*_IPTG_*-pbpP^+^* (EBT1241), *ΔsipP*/EV (EBT1244), *ΔsipP*/*P*_IPTG_*-pbpP^+^* (EBT1243), *ΔsipX ΔsipP*/EV (EBT1246), and *ΔsipX ΔsipP*/*P*_IPTG_*-pbpP^+^* (EBT1245). β-Galactosidase activities of cultures grown in the absence of IPTG are shown in Fig. S5A. (B) PbpP is produced in *ΔsipP* and *ΔsipX ΔsipP* mutants. The strains used are the same as described for panel A. After incubation to an OD of 1.6 to 1.8, 1 mL of each culture from panel A was concentrated, washed, and resuspended in Bocillin FL (50 μg/mL) for 30 min at room temperature. The color blot with both Bocillin FL and the ladder on a single gel is shown in Fig. S5B. Numbers on the left indicate the molecular masses (in kilodaltons) of the ladder. The band corresponding to PbpP is indicated on the right. (C) Overproduction of SipP can activate σ^P^ in the absence of PbpP. All strains contain *P_sigP_-lacZ* inserted into the *thrC* locus and the following relevant genotypes: WT/EV (EBT728), WT/*sipP*^+^ (EBT1269), *ΔpbpP*/EV (EBT1270), and *ΔpbpP*/*sipP*^+^ (EBT1273). Cultures grown in the absence of IPTG as well as overexpression of *bt0543* and *pbpP* are shown in Fig. S5C. (D) *sipP* overexpression is not sufficient for the cleavage of RsiP^S84W^ or RsiP^V82W^. All strains contain *P_sigP_-lacZ* and the following relevant genotypes: EV/EV (EBT1313), EV/*P*_IPTG_*-sipP* (EBT1314), *rsiP/EV* (EBT1323), *rsiP/P*_IPTG_*-sipP* (EBT1324), *rsiP^V82W^/EV* (EBT1319), *rsiP^V82W^/P*_IPTG_*-sipP* (EBT1320), *rsiP^S84W^/EV* (EBT1316), and *rsiP^S84W^/P*_IPTG_*-sipP* (EBT1317). Cultures grown in the absence of IPTG are shown in Fig. S5D. All strains were grown to mid-log phase (OD_600_, 0.6 to 0.8) in the presence of IPTG (1 mM) at 37°C. β-Galactosidase activities were calculated as described in Materials and Methods. Experiments were performed in technical and biological triplicates, and standard deviations are represented by error bars.

Previous work demonstrated that the penicillin-binding protein PbpP is required for σ^P^ activation (44). We also showed that overexpression of PbpP activates σ^P^ and hypothesized that PbpP senses β-lactams and activates σ^P^ by somehow controlling site 1 cleavage of RsiP (44). According to this model, PbpP acts upstream of RsiP cleavage by SipP. Therefore, overexpression of PbpP should have no effect on σ^P^ activation in the Δ*sipP* mutant. Consistently with this, we found that activation of σ^P^ by overexpression of PbpP required SipP in B. subtilis (Fig. 2B). In B. thuringiensis, we overexpressed *pbpP* from an IPTG-inducible promoter in the WT, *ΔsipX*, Δ*sipP*, and *ΔsipX* Δ*sipP* strains. Again, we found that the loss of SipX had no effect and that the overexpression of *pbpP* induced *P_sigP_-lacZ* expression in the absence of SipX (Fig. 5A). We found that Δ*sipP* blocked *P_sigP_-lacZ* expression when *pbpP* is overexpressed, as does the *ΔsipX ΔsipP* mutant (Fig. 5A). We concluded that SipP is required for PbpP to activate σ^P^.

10.1128/mbio.03707-21.7 FIG S5 IPTG Induction differences and color version for Fig. 5. *sipP* is required for activation of σ^P^ by overexpression of *pbpP*. (A) *pbpP* overexpression does not activate σ^P^ in the absence of *sipP*. All strains contain *P_sigP_-lacZ* inserted into the *thrC* locus and the following relevant genotypes: WT/EV (EBT1240), WT/*pbpP*^+^ (EBT1239), *ΔsipX*/EV (EBT1242), *ΔsipX*/*pbpP*^+^ (EBT1241), Δ*sipP*/EV (EBT1244), Δ*sipP*/*pbpP*^+^ (EBT1243), *ΔsipX* Δ*sipP*/EV (EBT1246), and *ΔsipX* Δ*sipP*/*pbpP*^+^ (EBT1245). (B) PbpP is produced in the Δ*sipP* and *ΔsipX* Δ*sipP* mutants (colored version). The strains used are the same as described for Fig. 5A. After incubation to an OD of 1.6 to 1.8, 1 mL of each culture from Fig. 5A was concentrated, washed, and resuspended in Bocillin FL (50 μg/mL) for 30 minutes at room temperature. Numbers on left indicate the molecular masses (in kilodaltons) of the ladder. The band corresponding to PbpP is indicated on the right. (C) All strains contain *P_sigP_-lacZ* inserted into the *thrC* locus and the following relevant genotypes: WT/EV (EBT1266), WT/*sipP*^+^ (EBT1269), WT/*bt0543*^+^ (EBT1160), Δ*pbpP*/EV (EBT1270), Δ*pbpP*/*bt0543*^+^ (EBT1257), and Δ*pbpP*/*sipP*^+^ (EBT1273). (D) *sipP* overexpression is not sufficient for cleavage of RsiP^S84W^ or RsiP^V82W^. All strains contain *P_sigP_-lacZ* and the following relevant genotypes: EV/EV (EBT1313), EV/*P*_IPTG_*-sipP* (EBT1314), *rsiP*/EV (EBT1323), *rsiP*/*P*_IPTG_*-sipP* (EBT1324), *rsiP^V82W^*/EV (EBT1319), *rsiP^V82W^*/*P*_IPTG_*-sipP* (EBT1320), *rsiP^S84W^*/EV (EBT1316), and *rsiP^S84W^*/*P*_IPTG_*-sipP* (EBT1317). All strains were grown to mid-log phase (OD_600_, 0.6 to 0.8) in the absence or presence of IPTG (1 mM) at 37°C. β-Galactosidase activities were calculated as described in Materials and Methods. Experiments were performed in technical and biological triplicates, and standard deviations are represented by error bars. Download FIG S5, TIF file, 1.8 MB. Copyright © 2022 Nauta et al. 2022 Nauta et al. https://creativecommons.org/licenses/by/4.0/ This content is distributed under the terms of the Creative Commons Attribution 4.0 International license.

To confirm that *pbpP* was being expressed similarly in all cells, we incubated the cultures from Fig. 5A with Bocillin FL, a fluorescent β-lactam that covalently binds PbpP. We observed a fluorescent band labeled with Bocillin FL approximately the size of PbpP in the samples that contained PbpP, and the band was absent in the empty-vector (EV) controls (Fig. 5B). Thus, PbpP is produced at levels similar to WT levels in the absence of SipP. We also observed lower Bocillin FL labeling of the WT and *ΔsipX* penicillin-binding proteins. As previously reported, this is due to degradation of Bocillin FL by β-lactamases that are transcribed when σ^P^ is activated by overexpression of *pbpP* (44).

Next, we sought to determine if PbpP is required for activation of σ^P^ when SipP is overproduced. To do this, we overexpressed *sipP* in WT and *ΔpbpP* cells. We found that overexpression of *sipP* in WT cells activated σ^P^ in the absence of cefoxitin (Fig. 5C). In Δ*pbpP* cells, overexpression of *sipP* activated σ^P^, but the β-galactosidase activity was ∼50 times lower than in WT cells (Fig. 5C). We also noted that overexpression of *sipP* leads to a subtle increase in *P_sigP_-lacZ* expression on X-Gal (5-bromo-4-chloro-3-indolyl-β-d-galactopyranoside) plates (Fig. S6). This suggests that SipP can cleave RsiP in the absence of PbpP; however, that cleavage is not efficient.

10.1128/mbio.03707-21.8 FIG S6 Overexpression of *sipP* activates σ^P^ in the absence of PbpP. All strains contain *P_sigP_-lacZ* inserted into the *thrC* locus and the following relevant genotypes: WT/EV (EBT1266), WT/*sipP^+^* (EBT1269), *ΔpbpP*/EV (EBT1270), and *ΔpbpP/sipP*^+^ (EBT1273). All strains were streaked on plates containing X-Gal (100 μg/mL), cam (10 μg/mL), and IPTG (1 mM). The plates were incubated overnight at 37°C. Download FIG S6, JPG file, 0.3 MB. Copyright © 2022 Nauta et al. 2022 Nauta et al. https://creativecommons.org/licenses/by/4.0/ This content is distributed under the terms of the Creative Commons Attribution 4.0 International license.

### Mutation of the predicted signal peptidase cleavage site prevents SipP cleavage of RsiP.

To ensure that SipP cleaved RsiP at relevant sites when it was overexpressed, we tested if SipP would activate σ^P^ in the presence of the RsiP signal peptidase cleavage site mutations (S84W, V82W). We constructed strains with *sipP* under the control of an IPTG-inducible promoter in a Δ*sigP-rsiP* mutant with *sigP^+^-rsiP^+^*, *sigP^+^-rsiP^S84W^*, or *sigP^+^-rsiP^V82W^* (Fig. 5D). We found that overexpression of *sipP* resulted in activation of σ^P^, but SipP was not able to activate σ^P^ when the signal peptidase cleavage site was mutated (Fig. 5D). We concluded that RsiP^V82W^ or RsiP^S84W^ cannot by cleaved by SipP. This suggests that SipP likely cleaves RsiP at the predicted signal peptidase cleavage site, VXS.

### Cefoxitin-induced σ^P^ activation in B. subtilis.

**FIG 6:**
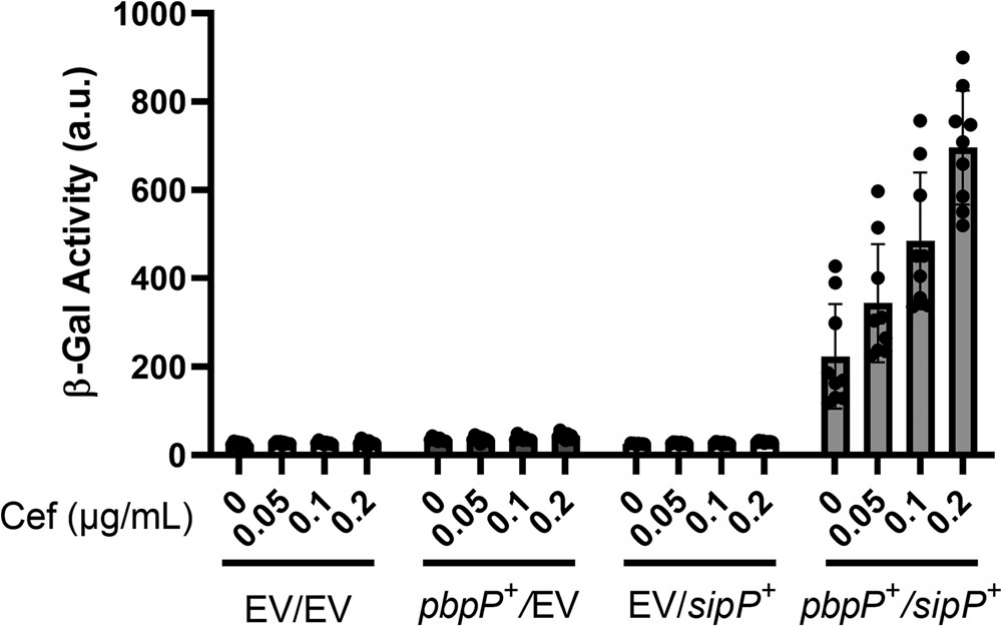
*pbpP* and *sipP* are sufficient for cefoxitin-induced activation of σ^P^ in B. subtilis. All strains are B. subtilis and contain *P_sigP-_sigP^+^-rsiP^+^-lacZ* and the following relevant genotypes: EV/EV (CDE3613), EV/*P_xyl_-pbpP^+^* (CDE3602), *P*_IPTG_*-sipP^+^*/EV (CDE3615), and *P*_IPTG_*-sipP^+^*/*P_xyl_-pbpP^+^* (CDE3610). All strains were grown to early mid-log phase (OD_600_, 0.6 to 0.8) in the presence of IPTG (0.01 mM) and xylose (0.01%) at 37°C. Cefoxitin (0.05 to 0.2 μg/mL) was added, and the cells were incubated for another hour at 37°C. β-Galactosidase activities were calculated as described in Materials and Methods. Experiments were performed in technical and biological triplicates, and standard deviations are represented by error bars.

In this and previous work, we have shown that PbpP, SipP, and RasP are required for σ^P^ (8, 44). To determine if their genes were sufficient for cefoxitin-induced σ^P^ activation, we constructed a B. subtilis strain containing all the known genes required for σ^P^ activation. B. subtilis does not contain a native copy of *sigP* and *rsiP*, so we integrated a copy of *sigP-rsiP* under its native promoter. We then introduced *pbpP* (*P_xyl_-pbpP*) and *sipP* (*P*_IPTG_*-sipP*) genes into the chromosome. We relied on the endogenous B. subtilis *rasP* gene for site 2 protease activity. When both PbpP and SipP were present, we found that σ^P^ was activated in response to cefoxitin in a dose-dependent manner (Fig. 6). In the absence of either PbpP or SipP, σ^P^ is not activated in B. subtilis (Fig. 6). Combined with our previous work, these data suggest that the presence *sigP*, *rsiP*, *pbpP*, *sipP*, and *rasP* genes is sufficient for sensing cefoxitin, inducing RsiP degradation, and allowing activation of σ^P^.

### Bacillus anthracis contains a mutation in *rsiP* that blocks site 1 cleavage.

β-Lactam sensitivity has been one of the defining features that differentiates most strains of B. anthracis from B. cereus or B. thuringiensis (52, 53). However, β-lactam-resistant strains of B. anthracis have been reported (43, 45, 46). Interestingly, both β-lactam-sensitive and -resistant B. anthracis strains carry all of the known components necessary for activation of σ^P^, including *sigP*, *rsiP*, *rasP*, *sipP*, and *pbpP*. The resistant strains have mutations in *rsiP* that result in constitutive σ^P^ activation and therefore constitutive expression of β-lactamases (43, 45, 46). The sensitive strains of B. anthracis, while carrying *sigP-rsiP*, do not respond to β-lactams (43). Previous work demonstrated that B. anthracis likely contains the genes required for σ^P^ activation, because a *sigP-rsiP* mutant complemented with *sigP-rsiP* from B. thuringiensis responds to β-lactams (43). The authors concluded that *rsiP* from B. anthracis likely contains a mutation that prevents it from responding to β-lactams and activating σ^P^. We aligned the RsiP amino acid sequences from B. anthracis and B. thuringiensis to identify changes that may result in unresponsive B. anthracis RsiP. While the proteins are 88% identical and 93% similar, we observed an amino acid change in the signal peptidase cleavage site of B. anthracis RsiP (Fig. S1B). This sequence, VQI (rather than VQS, as in B. thuringiensis and B. cereus), is not recognized by SignalP as a signal peptidase cleavage site (Fig. S1B). Based on these observations, we sought to determine if this single amino acid difference prevents B. anthracis RsiP degradation in response to β-lactams *in vivo*.

**FIG 7:**
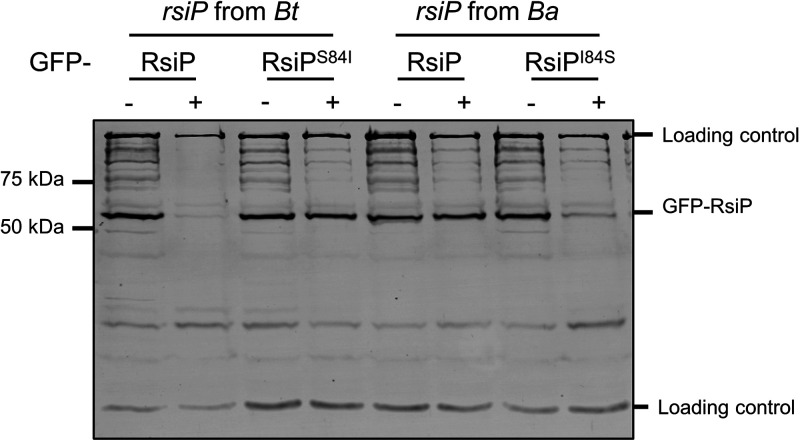
The signal peptidase cleavage site in RsiP from B. anthracis blocks RsiP degradation. All strains contain the following relevant genotypes: GFP-RsiP*_Bt_* (EBT936), GFP-RsiP^S84I^*_Bt_* (EBT1328), GFP-RsiP*_Ba_* (EBT1329), and GFP-RsiP^I84S^*_Ba_* (EBT1330). Cells were grown to mid-log phase (OD_600_, 0.6 to 0.8) with 1 mM IPTG at 37°C, pelleted, and concentrated in LB or LB plus cefoxitin (5 μg/mL). Cells were incubated for 1 h at 37°C before sample buffer was added. Immunoblotting was performed using anti-GFP antisera. Streptavidin IR680LT was used to detect PycA (HD73_4231) and AccB (HD73_4487), which served as loading controls (68, 69). A color blot with both anti-GFP and streptavidin on a single gel is shown in Fig. S7. Numbers at the right indicate the molecular masses (in kilodaltons) of the ladder.

We expressed *gfp-rsiP* from B. thuringiensis (*gfp-rsiP_Bt_*), *gfp-rsiP^S84^_Bt_*, *gfp-gfp-rsiP* from B. anthracis (*rsiP_Ba_*), and *gfp-rsiP^I84S^_Ba_* in WT B. thuringiensis (Fig. 7). As previously observed, WT GFP-RsiP*_Bt_* was degraded in response to cefoxitin (Fig. 7). Interestingly, the B. thuringiensis RsiP^S84I^ mutant prevented RsiP degradation in response to cefoxitin (Fig. 7). We found that WT GFP-RsiP*_Ba_* was not degraded in response to cefoxitin, but GFP-RsiP^I84S^*_Ba_* was degraded in response to cefoxitin (Fig. 7). From these data, we conclude that isoleucine at position 84 blocks site 1 cleavage of RsiP by signal peptidases and thus blocks σ^P^ activation. We hypothesize that this mutation prevents signal peptidases from recognizing the RsiP cleavage site. We also hypothesize that the mutation of the RsiP cleavage site explains why most B. anthracis strains fail to activate σ^P^ and are sensitive to β-lactams.3.

10.1128/mbio.03707-21.9 FIG S7 Color version of Fig. 7. The signal peptidase cleavage site in RsiP from B. anthracis blocks σ^P^ activation. All strains contain the following relevant genotypes: GFP-RsiP*_Bt_* (EBT1327), GFP-RsiP^S84I^*_Bt_* (EBT1328), GFP-RsiP*_Ba_* (EBT1329), and GFP-RsiP^I84S^*_Ba_* (EBT1330). Cells were grown to mid-log phase (OD_600_, 0.6 to 0.8) with 1 mM IPTG at 37°C, pelleted, and concentrated in LB or LB plus cefoxitin (5 μg/mL). Cells were incubated for 1 h at 37°C before sample buffer was added. Immunoblotting was performed using anti-GFP antisera. Streptavidin IR680LT was used to detect PycA (HD73_4231) and AccB (HD73_4487), which served as loading controls (68, 69). Numbers at the right indicate the molecular masses (in kilodaltons) of the ladder. Download FIG S7, TIF file, 2.3 MB. Copyright © 2022 Nauta et al. 2022 Nauta et al. https://creativecommons.org/licenses/by/4.0/ This content is distributed under the terms of the Creative Commons Attribution 4.0 International license.

## DISCUSSION

In summary, the data presented here suggest that the signal peptidase SipP is responsible for cleaving RsiP at site 1 in response to β-lactams. This conclusion is supported by several pieces of data. First, we identified a predicted signal peptidase cleavage site and showed that mutation of critical residues (V82W and S84W) blocked RsiP degradation. Second, we conducted a screen and determined that the B. thuringiensis signal peptidases *sipP* and *bt0543* are capable of cleaving RsiP in B. subtilis. Third, we found that deletion of *sipP* in B. thuringiensis resulted in blocked RsiP cleavage at site 1, while deletion of *bt0543* had no effect on RsiP degradation. Together, these data suggest that SipP is the site 1 protease required for σ^P^ activation.

### SipP is the site 1 protease required for σ^P^ activation.

Anti-σ factors that are degraded to release their cognate ECF σ factor are cleaved by a site 1 protease at site 1 and subsequently by a site 2 protease at site 2 (54, 55). We previously showed that the site 2 protease RasP is required for RsiP degradation and σ^P^ activation (8). Here, we show that overexpression of either SipP or SipX in either B. subtilis or B. thuringiensis led to constitutive activation of σ^P^, presumably by cleaving RsiP at site 1 (Fig. 1A). We found that deletion of *sipP* in B. thuringiensis blocked σ^P^ activation and that expressing *sipP* at low levels restored cefoxitin-induced σ^P^ activation. This demonstrated that the loss of σ^P^ activation in the *sipP* deletion strain is due to a loss of *sipP*. We also found that the loss of SipP resulted in a loss of RsiP degradation in response to cefoxitin, suggesting that SipP is required for the cleavage of RsiP at site 1. Disruption of the putative signal peptidase cleavage site blocked site 1 cleavage of RsiP (Fig. 1B and C). Importantly, overexpression of SipP does not lead to cleavage of RsiP if the signal peptidase cleavage site is mutated. Therefore, SipP likely cleaves RsiP after the VQS signal peptidase motif (Fig. 5D).

We identified that a single point mutation in the *rsiP* gene of B. anthracis renders it unresponsive to β-lactams (Fig. 7). This mutation likely alters in the signal peptidase cleavage site so that it cannot be recognized by SipP. We showed that changing the B. anthracis RsiP cleavage site (VQI) to the B. thuringiensis RsiP sequence (VQS) restored RsiP degradation. We also showed that changing the B. thuringiensis cleavage site (VQS) to the B. anthracis sequence (VQI) resulted in a loss of RsiP degradation. Thus, we have concluded that many B. anthracis stains are likely sensitive to β-lactams because signal peptidases fail to cleave RsiP at site 1 in response to β-lactams.

While we found that SipX can activate σ^P^ when overexpressed, deletion of *sipX* has no effect on σ^P^ activation, β-lactam MIC, or RsiP degradation. This leads us to conclude that SipX does not play a physiologic role in σ^P^ activation in B. thuringiensis. In addition, the presence or absence of PbpP did not alter activation of σ^P^ by overexpression of SipX. These data further suggest that SipX cleavage of RsiP is an artifact of overexpression.

We found that overexpression of SipP in the wild type increases the expression of *P_sigP_-lacZ* by >1,000-fold; however, in the absence of PbpP, SipP overexpression increased *P_sigP_-lacZ* expression only ∼5-fold (Fig. 5C). This is consistent with previous work showing that PbpP is required for RsiP degradation at site 1 (44). Our data demonstrate that SipP is the site 1 protease responsible for cefoxitin-induced cleavage of RsiP. Reconstitution of the σ^P^ signaling system in B. subtilis suggests that we have identified all the unique B. thuringiensis genes required for σ^P^ activation.

### Model for σ^P^ activation.

**FIG 8:**
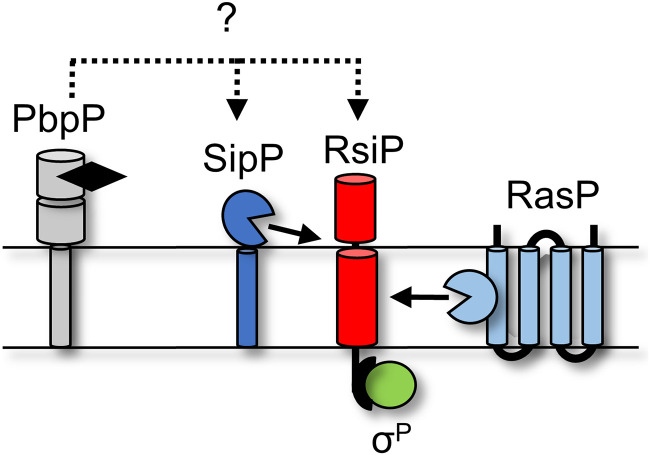
Model for σ^P^ activation. PbpP (gray) binds β-lactams (black) and conformationally changes. This allows a direct or indirect interaction between RsiP (red) or SipP (dark blue). This interaction results in site 1 cleavage of RsiP (red) by SipP (dark blue). Site 1 cleavage allows for site 2 cleavage by RasP (light blue). Degradation of RsiP (red) results in the release of σ^P^ (green).

The data presented here and in our previous work support the following model: (i) PbpP binds β-lactams and interacts with either RsiP or SipP, (ii) SipP cleaves RsiP at site 1, (iii) RasP cleaves RsiP at site 2, (iv) σ^P^ is activated and induces the expression of its regulon, which includes PBPs and β-lactamases, and (v) the β-lactamases then degrade the β-lactams (Fig. 8).

Because increased *sipP* expression induces σ^P^ activation, we considered the possibility that *sipP* expression is induced by β-lactams and that this increased expression allows it to cleave RsiP. However, we found that when *sipP* expression was uncoupled from its native promoter and expressed under the control of an IPTG-inducible promoter, σ^P^ was still activated in a dose-dependent manner by cefoxitin (Fig. 6). This suggests that induction of *sipP* transcription by cefoxitin is likely not required to activate σ^P^ (Fig. 5D). Thus, the dose-dependent increase in σ^P^ activation in response to cefoxitin must be attributed to another factor, likely regulation of SipP activity by PbpP.

We previously established that activation of σ^P^ required the presence of the penicillin-binding protein PbpP and that the absence of PbpP blocked site 1 cleavage of RsiP (44). We found that when overexpressed, SipP can cleave RsiP in the absence of β-lactams (Fig. 5C and Fig. S6). However, overexpression of PbpP enhances the activation of σ^P^ to a much larger degree (Fig. 2B and 5C; Fig. S5C). Importantly we found that overexpression of PbpP does not lead to σ^P^ activation in the absence of *sipP* (Fig. 5A). Thus, activation of σ^P^ by overproduction of PbpP is dependent on SipP, but SipP cleavage, while affected by PbpP, is not entirely dependent on PbpP. This suggests that SipP acts downstream of PbpP (Fig. 8).

We hypothesize that the likely model for σ^P^ activation involves PbpP binding to β-lactams, which promotes an interaction either directly or indirectly with SipP. SipP then cleaves RsiP at site 1, initiating the proteolytic destruction of RsiP and σ^P^ activation. In support of this model, we find that RsiP^S84A^ is constitutively degraded in B. thuringiensis, suggesting that the cleavage site is accessible and likely not occluded in the absence of stress (Fig. S8). Unfortunately, to date we have been unable to detect an interaction between PbpP and SipP by two-hybrid assays, and PbpP has been difficult to purify (44). Future work will determine which proteins directly interact to control site 1 cleavage of RsiP and thus σ^P^ activation.

10.1128/mbio.03707-21.10 FIG S8 Degradation of RsiP^S84A^ is independent of SipP and PbpP. Strains have the following relevant genotypes: GFP-RsiP (EBT936), GFP-RsiP^S84A^ (EBT1208), Δ*sipP*/GFP-RsiP (EBT1222), Δ*sipP*/GFP-RsiP^S84A^ (EBT1302), Δ*pbpP*/GFP-RsiP (EBT937), and Δ*pbpP*/GFP-RsiP^S84A^ (EBT1301). Cells were grown to mid-log phase (OD_600_, 0.6 to 0.8) with 1 mM IPTG at 37°C. They were pelleted and concentrated in LB or LB plus cefoxitin (5 μg/mL). Cells were incubated for 1 h at 37°C before sample buffer was added. Immunoblotting was performed using anti-GFP antisera. Streptavidin IR680LT was used to detect PycA (HD73_4231) and AccB (HD73_4487), which served as loading controls (68, 69). Numbers at the right indicate the molecular masses (in kilodaltons) of the ladder. Download FIG S8, JPG file, 0.2 MB. Copyright © 2022 Nauta et al. 2022 Nauta et al. https://creativecommons.org/licenses/by/4.0/ This content is distributed under the terms of the Creative Commons Attribution 4.0 International license.

### A growing role for signal peptidases in ECF σ factor activation.

Type I signal peptidases are membrane-bound serine proteases that cleave the leader peptide of secreted proteins, thus freeing them from the membrane and allowing secretion or localization to the periplasm or outer membrane (37, 56). SipS and SipT are the major type I signal peptidases in B. subtilis and are redundant and essential (39). Activation of σ^V^ occurs when RsiV binds lysozyme and reveals a signal peptidase, which is cleaved by SipS or SipT (6, 31, 36). The signal peptidase cleavage site is embedded in an amphipathic helix that is part of a DUF4179 domain (36). RsiP does not contain the DUF4179 domain, nor does it have an amphipathic helix, suggesting that site 1 cleavage may be controlled by a different mechanism.

The major signal peptidases of B. subtilis are not sufficient to cleave RsiP (Fig. 1A). The canonical signal peptidase cleavage site is AXA, while the cleavage site in RsiP is VQS (38). In addition, B. thuringiensis has more signal peptidases than B. subtilis, raising the possibility that they may recognize different cleavage sites. This is supported by our finding that mutation of S84 to an alanine results in constitutive degradation of RsiP (Fig. 1C). In fact, degradation of RsiP^S84A^ appears constitutive even in the absence of PbpP and SipP (Fig. S8). We concluded that mutating VQS to VQA made the RsiP cleavage site recognizable by other signal peptidases in B. thuringiensis. This supports the hypothesis that B. thuringiensis encodes signal peptidases that have different signal peptidase cleavage site specificities. Future work will be required to determine if only SipP cleaves RsiP, if it is involved in the secretion of other proteins, or if it plays additional roles in cell signaling or homeostasis.

## MATERIALS AND METHODS

### Media and growth conditions.

All B. thuringiensis strains are isogenic derivatives of AW43, a derivative of B. thuringiensis serovar kurstaki strain HD73 (57). All B. subtilis strains are derivatives of 168 or PY79 (58). The strains and genotypes can be found in Table 3. All B. thuringiensis strains were grown in or on LB medium at 30°C unless otherwise specified. Liquid cultures of B. thuringiensis were grown with agitation in a roller drum. B. thuringiensis strains containing episomal plasmids were grown in LB medium containing erythromycin (erm, 10 μg/mL; Amresco). E. coli strains were grown at 37°C using LB-ampicillin (amp, 100 μg/mL; Amresco) or LB-chloramphenicol (cam, 10 μg/mL; Amresco) medium. B. subtilis strains were grown on LB with antibiotics (cam, 10 μg/mL; spectinomycin [spec], 100 μg/mL [Amresco]; or erm, 10 μg/mL). The β-galactosidase chromogenic indicator 5-bromo-4-chloro-3-indolyl β-d-galactopyranoside (X-Gal; Research Products International) was used at a concentration of 100 μg/mL. IPTG (Research Products International) and xylose (Acros) were used at the concentrations indicated in the figure legends. Cefoxitin (Sigma-Aldrich) was used at the concentrations listed in the figure legends.

**TABLE 3:** Strains

Strain	Description	Reference or source
B. thuringiensis		
AW43	B. thuringiensis serovar kurstaki HD73 cured of both pAW63 and pHT73, Nal^r^	57
EBT251	AW43 *thrC*::*P_sigP_-lacZ ΔsigP-rsiP*/pAH9	8
EBT238	AW43 *thrC*::*P_sigP_-lacZ ΔsigP-rsiP*/pTHE690 (pAH9 *P_sigP_ sigP-rsiP*)	8
EBT1136	AW43 *thrC*::*P_sigP_-lacZ ΔsigP-rsiP*/pCDE832 (pAH9 *P_sigP_ sigP-rsiP^S84W^*)	This study
EBT1165	AW43 *thrC*::*P_sigP_-lacZ ΔsigP-rsiP*/pCDE846 (pAH9 *P_sigP_ sigP-rsiP^V82W^*)	This study
EBT1166	AW43 *thrC*::*P_sigP_-lacZ ΔsigP-rsiP*/pCDE851 (pAH9 *P_sigP_ sigP-rsiP^S84A^*)	This study
EBT936	AW43 *thrC*::*P_sigP_*-*lacZ* ICE*Bs1*::*P*_IPTG_*-gfp-rsiP tetM cat*	44
EBT1207	AW43 *thrC*::*P_sigP_-lacZ* ICE*Bs1*::*P*_IPTG_*-gfp-rsiP*^S84W^ *tetM cat*	This study
EBT1209	AW43 *thrC*::*P_sigP_-lacZ* ICE*Bs1*::*P*_IPTG_*-gfp-rsiP*^V82W^ *tetM cat*	This study
EBT1208	AW43 *thrC*::*P_sigP_-lacZ* ICE*Bs1*::*P*_IPTG_*-gfp-rsiP*^S84A^ *tetM cat*	This study
EBT939	AW43 *thrC*::*P_sigP_-lacZ ΔrasP* ICE*Bs1*::*P*_IPTG_*-gfp-rsiP tetM cat*	44
EBT1210	AW43 *thrC*::*P_sigP_-lacZ ΔrasP* ICE*Bs1*::*P*_IPTG_*-gfp-rsiP*^S84W^ *tetM cat*	This study
EBT1212	AW43 *thrC*::*P_sigP_-lacZ ΔrasP* ICE*Bs1*::*P*_IPTG_*-gfp-rsiP*^V82W^ *tetM cat*	This study
EBT1211	AW43 *thrC*::*P_sigP_-lacZ ΔrasP* ICE*Bs1*::*P*_IPTG_*-gfp-rsiP*^S84A^ *tetM cat*	This study
THE2549	AW43 *thrC*::*P_sigP_-lacZ*	44
EBT1170	AW43 *thrC*::*P_sigP_-lacZ ΔsipX*	This study
EBT1202	AW43 *thrC*::*P_sigP_-lacZ ΔsipP*	This study
EBT1213	AW43 *thrC*::*P_sigP_-lacZ ΔsipX ΔsipP*	This study
EBT1223	AW43 *thrC*::*P_sigP_-lacZ ΔsipX* ICE*Bs1*::*P*_IPTG_*-gfp-rsiP tetM cat*	This study
EBT1222	AW43 *thrC*::*P_sigP_-lacZ ΔsipP* ICE*Bs1*::*P*_IPTG_*-gfp-rsiP tetM cat*	This study
EBT1224	AW43 *thrC*::*P_sigP_-lacZ ΔsipX ΔsipP* ICE*Bs1*::*P*_IPTG_*-gfp-rsiP tetM cat*	This study
EBT1265	AW43 *thrC*::*P_sigP_-lacZ ΔsipX ΔsipP ΔrasP* ICE*Bs1*::*P*_IPTG_*-gfp-rsiP tetM cat*	This study
EBT728	AW43 *thrC*::*P_sigP_-lacZ* ICE*Bs1*::EV *tetM cat*	44
EBT1239	AW43 *thrC*::*P_sigP_-lacZ* ICE*Bs1*::*P*_IPTG_*-pbpP tetM cat*	44
EBT1160	AW43 *thrC*::*P_sigP_-lacZ* ICE*Bs1*::*P*_IPTG_*-sipX tetM cat*	This study
EBT1263	AW43 *thrC*::*P_sigP_-lacZ ΔsipX ΔrasP* ICE*Bs1*::*P*_IPTG_*-gfp-rsiP tetM cat*	This study
EBT1242	AW43 *thrC*::*P_sigP_-lacZ ΔsipX* ICE*Bs1*::EV *tetM cat*	This study
EBT1241	AW43 *thrC*::*P_sigP_-lacZ ΔsipX* ICE*Bs1*::*P*_IPTG_*-pbpP tetM cat*	This study
EBT1244	AW43 *thrC*::*P_sigP_-lacZ ΔsipP* ICE*Bs1*::EV *tetM cat*	This study
EBT1243	AW43 *thrC*::*P_sigP_-lacZ ΔsipP* ICE*Bs1*::*P*_IPTG_*-pbpP tetM cat*	This study
EBT1218	AW43 *thrC*::*P_sigP_-lacZ ΔsipP* ICE*Bs1*::*P*_IPTG_*-sipP tetM cat*	This study
EBT1246	AW43 *thrC*::*P_sigP_-lacZ ΔsipX ΔsipP* ICE*Bs1*::EV *tetM cat*	This study
EBT1245	AW43 *thrC*::*P_sigP_-lacZ ΔsipX ΔsipP* ICE*Bs1*::*P*_IPTG_*-pbpP tetM cat*	This study
EBT1220	AW43 *thrC*::*P_sigP_-lacZ ΔsipX ΔsipP* ICE*Bs1*::*P*_IPTG_*-sipP tetM cat*	This study
EBT1269	AW43 *thrC*::*P_sigP_-lacZ* ICE*Bs1*::*P*_IPTG_*-sipP tetM cat*	This study
EBT1270	AW43 *thrC*::*P_sigP_-lacZ ΔpbpP* ICE*Bs1*::EV *tetM cat*	This study
EBT1273	AW43 *thrC*::*P_sigP_-lacZ ΔpbpP* ICE*Bs1*::*P*_IPTG_*-sipP tetM cat*	This study
EBT1257	AW43 *thrC*::*P_sigP_-lacZ ΔpbpP* ICE*Bs1*::*P*_IPTG_*-sipX tetM cat*	This study
EBT1313	AW43 *thrC*::*P_sigP_-lacZ ΔsipP rsiP* ICE*Bs1*::EV *tetM cat*/pAH9	This study
EBT1314	AW43 *thrC*::*P_sigP_-lacZ ΔsipP rsiP* ICE*Bs1*::*P*_IPTG_*_-_sipP tetM cat*/pAH9	This study
EBT1323	AW43 *thrC*::*P_sigP_-lacZ ΔsipP rsiP* ICE*Bs1*::EV *tetM cat*/pTHE960 (*P_sigP_-sigP-rsiP*)	This study
EBT1324	AW43 *thrC*::*P_sigP_-lacZ ΔsipP rsiP* ICE*Bs1*::*P*_IPTG_*-sipP tetM cat*/pTHE960 (*P_sigP_-sigP-rsiP*)	This study
EBT1319	AW43 *thrC*::*P_sigP_-lacZ ΔsipP rsiP* ICE*Bs1*::EV *tetM cat*/pCDE832 (*P_sigP_-sigP-rsiP^V82W^*)	This study
EBT1320	AW43 *thrC*::*P_sigP_-lacZ ΔsipP rsiP* ICE*Bs1*::*P*_IPTG_*-sipP tetM cat*/pCDE832 (*P_sigP_-sigP-rsiP^V82W^*)	This study
EBT1316	AW43 *thrC*::*P_sigP_-lacZ ΔsipP rsiP* ICE*Bs1*::EV *tetM cat*/pCDE846 (*P_sigP_-sigP-rsiP^S84W^*)	This study
EBT1317	AW43 *thrC*::*P_sigP_-lacZ ΔsipP rsiP* ICE*Bs1*::*P*_IPTG_*-sipP tetM cat*/pCDE846 (*P_sigP_-sigP-rsiP^S84W^*)	This study
EBT1328	AW43 *thrC*::*P_sigP_-lacZ* ICE*Bs1*::*P*_IPTG_*-gfp-rsiP*^S84I^ *tetM cat*	This study
EBT1329	AW43 *thrC*::*P_sigP_-lacZ* ICE*Bs1*::*P*_IPTG_*-gfp-rsiP* (B. anthracis *Sterne*) *tetM cat*	This study
EBT1330	AW43 *thrC*::*P_sigP_-lacZ* ICE*Bs1*::*P*_IPTG_*-gfp-rsiP^I84S^* (B. anthracis *Sterne*) *tetM cat*	This study
EBT1302	AW43 *thrC*::*P_sigP_-lacZ ΔsipP* ICE*Bs1*::*P*_IPTG_*-gfp-rsiP^S84A^ tetM cat*	This study
EBT937	AW43 *thrC*::*P_sigP_-lacZ ΔpbpP* ICE*Bs1*::*P*_IPTG_*-gfp-rsiP tetM cat*	This study
EBT1301	AW43 *thrC*::*P_sigP_-lacZ ΔpbpP* ICE*Bs1*::*P*_IPTG_*-gfp-rsiP^S84A^ tetM cat*	This study

B. subtilis		
PY79	Prototrophic derivative of B. subtilis 168	58
CDE3602	PY79 *thrC*::*P_sigP_-sigP^+^-rsiP^+^-lacZ erm amyE*::*P_xyl_-pbpP^+^ spec* ICE*Bs1*::*P*_IPTG_*-tetM cat*	This study
CDE3603	PY79 *thrC*::*P_sigP_-sigP^+^-rsiP^+^-lacZ erm amyE*::*P_xyl_-pbpP^+^ spec* ICE*Bs1*::*P*_IPTG_*-bt2887 tetM cat*	This study
CDE3604	PY79 *thrC*::*P_sigP_-sigP^+^ rsiP^+^-lacZ erm amyE*::*P_xyl_-pbpP^+^ spec* ICE*Bs1*::*P*_IPTG_*-sipX tetM cat*	This study
CDE3605	PY79 *thrC*::*P_sigP_-sigP^+^ rsiP^+^-lacZ erm amyE*::*P_xyl_-pbpP^+^ spec* ICE*Bs1*::*P*_IPTG_*-bt1507 tetM cat*	This study
CDE3606	PY79 *thrC*::*P_sigP_-sigP^+^ rsiP^+^-lacZ erm amyE*::*P_xyl_-pbpP^+^ spec* ICE*Bs1*::*P*_IPTG_*-bt2973 tetM cat*	This study
CDE3608	PY79 *thrC*::*P_sigP_-sigP^+^-rsiP^+^-lacZ erm amyE*::*P_xyl_-pbpP^+^ spec* ICE*Bs1*::*P*_IPTG_*-bt3371 tetM cat*	This study
CDE3610	PY79 *thrC*::*P_sigP_-sigP^+^-rsiP^+^-lacZ erm amyE*::*P_xyl_-pbpP^+^ spec* ICE*Bs1*::*P*_IPTG_*-sipP tetM cat*	This study
CDE3612	PY79 *thrC*::*P_sigP_-sigP^+^-rsiP^+^-lacZ erm amyE*::*P_xyl_-pbpP^+^ spec* ICE*Bs1*::*P*_IPTG_*-bt2898 tetM cat*	This study
CDE3613	PY79 *thrC*::*P_sigP_-sigP^+^-rsiP^+^-lacZ erm* ICE*Bs1*::*P*_IPTG_*-tetM cat*	This study
CDE3614	PY79 *thrC*::*P_sigP_-sigP^+^-rsiP^+^-lacZ erm* ICE*Bs1*::*P*_IPTG_*-sipX tetM cat*	This study
CDE3615	PY79 *thrC*::*P_sigP_-sigP^+^-rsiP^+^-lacZ erm* ICE*Bs1*::*P*_IPTG_*-sipP tetM cat*	This study

E. coli		
OmniMAX 2 T1R	F′ {*proAB^+^ lacI*^q^ *lacZΔ*M15 Tn*10 (*Tet^r^) Δ(*ccdAB*)} *mcrA Δ*(*mrr-hsdRMS-mcrBC*) ϕ80(*lacZ*)*Δ*M15 *Δ*(*lacZYA-argF*)*U169 endA1 recA1 supE44 thi-1 gyrA96 relA1 tonA panD*	Invitrogen
INV110	*endA1 rpsL thr leu thi lacY galK galT ara tomA tsx dam dcm supE44* Δ(*lac-proAB*) [F' *traD36 proAB lacI*^q^ZΔM15]	Invitrogen

### Strain and plasmid construction.

All plasmids are listed in Table 4 and Table S1 in the supplemental material, which include additional information relevant to plasmid assembly. Plasmids were constructed by isothermal assembly (New England Biolabs) (59). Regions of plasmids constructed using PCR were verified by DNA sequencing. The oligonucleotide primers used in this work were synthesized by Integrated DNA Technologies (Coralville, IA) and are listed in Table S2. All plasmids were propagated using OmniMAX 2 T1R as the cloning host and passaged through the nonmethylating E. coli strain INV110 before being transformed into a B. thuringiensis recipient strain.

**TABLE 4:** Plasmids

Plasmid	Relevant features	Reference or source
pMAD	*ori*-pE194ts *amp erm*	60
pAH9	*ori*-pE194 *P_sarA_-mCherry amp erm*	70
pDR160	*amyE*::*P_xyl_ amp spec*	David Rudner
pDG1663	*thrC*::*lacZ erm amp*	71
pDR111	*amyE*::*P*_IPTG_ *amp spec*	David Rudner
pCE695	*amyE*::*P*_IPTG_-*gfp-rsiP amp spec*	44
pJAB980	ICE::*P*_IPTG_*-gfp amp cat*	61
pCE697	ICE*Bs1*::*P*_IPTG_ *amp cat*	44
pCE698	ICE*Bs1*::*P*_IPTG_-*gfp-rsiP amp cat*	44
pCE707	ICE::*P*_IPTG_*-pbpP^+^ amp cat*	44
pTHE960	*ori*-pE194 *P_sigP_-sigP^+^ rsiP^+^ amp erm*	8
pCE868	ICE*Bs1*::*P*_IPTG_-*gfp-rsiP^S84W^ amp cat*	This study
pCE869	ICE*Bs1*::*P*_IPTG_-*gfp-rsiP^V82W^ amp cat*	This study
pCE870	ICE*Bs1*::*P*_IPTG_-*gfp-rsiP^S84A^ amp cat*	This study
pCE832	*ori*-pE194 *P_sigP_-sigP^+^rsiP^S84W^ amp erm*	This study
pCE846	*ori*-pE194 *P_sigP_-sigP^+^rsiP^V82W^ amp erm*	This study
pCE851	*ori*-pE194 *P_sigP_-sigP^+^rsiP^S84A^ amp erm*	This study
pCE834	ICE::*P*_IPTG_*-sipX ^+^ amp cat*	This study
pCE835	ICE::*P*_IPTG_*-bt1507^+^ amp cat*	This study
pCE833	ICE::*P*_IPTG_*-bt2887^+^ amp cat*	This study
pCE847	ICE::*P*_IPTG_*-bt2898^+^ amp cat*	This study
pCE836	ICE::*P*_IPTG_*-bt2973^+^ amp cat*	This study
pCE838	ICE::*P*_IPTG_*-bt3371^+^ amp cat*	This study
pCE840	ICE::*P*_IPTG_*-sipP^+^* (*bt4122) amp cat*	This study
pCE897	ICE*Bs1*::*P*_IPTG_-*gfp-rsiP^S84I^ amp cat*	This study
pCE905	ICE*Bs1*::*P*_IPTG_-*gfp-rsiP* (B. anthracis Sterne) *amp cat*	This study
pCE906	ICE*Bs1*::*P*_IPTG_-*gfp-rsiP^I84S^* (B. anthracis Sterne) *amp cat*	This study
pCE852	Δ*bt0543 ori*-pE194^ts^ *amp erm*	This study
pCE853	Δ*sipP ori*-pE194^ts^ *amp erm*	This study
pCE795	*amyE*::*P_xyl_*-*pbpP*^+^ *amp spec*	This study
pCE811	*thrC*::*P_sigP_-sigP^+^ rsiP^+^-lacZ erm amp*	This study

10.1128/mbio.03707-21.1 TABLE S1 Plasmids used in this study. Download Table S1, PDF file, 0.1 MB. Copyright © 2022 Nauta et al. 2022 Nauta et al. https://creativecommons.org/licenses/by/4.0/ This content is distributed under the terms of the Creative Commons Attribution 4.0 International license.

10.1128/mbio.03707-21.2 TABLE S2 Primers used in this study. Download Table S2, PDF file, 0.1 MB. Copyright © 2022 Nauta et al. 2022 Nauta et al. https://creativecommons.org/licenses/by/4.0/ This content is distributed under the terms of the Creative Commons Attribution 4.0 International license.

To construct deletion mutants, we cloned ∼1 kb of DNA upstream and 1 kb downstream of the site of the desired deletion using primers listed in Table S2 onto the temperature-sensitive (erythromycin-resistant) pMAD plasmid between the BglII and EcoRI sites (60). Mutants were constructed by shifting temperatures as previously described (60).

The plasmids carrying IPTG-inducible signal peptidases were constructed by amplifying the open reading frame of each gene using the primers listed in Table S2. The resulting PCR products were then cloned into pCE697, digested with SalI and NheI (44).

The plasmids carrying GFP-RsiP and mutant versions were constructed by amplifying the open reading frame of each gene using the primers listed in Table S2. The resulting PCR products were then cloned into pCE697, digested with SalI and NheI (44).

B. subtilis ICE*Bs1* conjugation strains were constructed by transforming JAB932 as previously described (61, 62). The resulting transformants or donor strains were grown in LB with d-alanine (100 μg/mL) for 2 h, at which point 1% xylose was added and cells were grown for 1 h. Recipient strains of B. thuringiensis were grown to an optical density at 600 nm (OD_600_) of ∼0.8. The donor and recipient strains were mixed at equal concentrations, plated on LB plus d-alanine (100 μg/mL), and incubated for 6 h. Transconjugants were isolated by plating on LB plus chloramphenicol plates.

### B. thuringiensis DNA transformation.

Plasmids were introduced into B. thuringiensis by electroporation (63, 64). Briefly, recipient cells were grown to late log phase at 37°C from a fresh plate. For each transformation, cells (1.5 mL) were pelleted by centrifugation (8,000 rpm) and washed twice in room temperature sterile water. After careful removal of all residual water, 100 μl of filter-sterilized 40% polyethylene glycol 6000 (PEG 6000; Sigma) was used to gently resuspend cells. Approximately 2 to 10 μl of unmethylated DNA (>50 ng/μL) was added to cells and transferred to a 0.4-cm-gap electroporation cuvette (Bio-Rad). Cells were exposed to 2.5 kV for 4 to 6 ms. LB was immediately added, and cells were incubated at 30°C for 1 to 2 h prior to being plated on selective media.

### β-Galactosidase assays.

To quantify expression from the *sigP* promoter, we measured the β-galactosidase activity of cells containing a *P_sigP_*-*lacZ* promoter fusion. Overnight cultures were diluted 1:50 in fresh LB media and incubated to mid-log phase (OD of 0.6 to 0.8) at 30°C with 1% xylose or 1 mM IPTG. Antibiotics were added to 1 mL of each subculture at the concentrations listed. Cells were incubated for 1 h at 37°C with agitation. If antibiotics were not used for activation of σ^P^, the cultures were grown to mid-log phase (0.6 to 0.8) at 37°C with IPTG and/or xylose. The cultures were then grown for another hour to an OD_600_ of 1.6 to 1.8 at 37°C with agitation. One milliliter of each sample was pelleted and resuspended in 1 mL of Z-buffer (16.1 g/L Na_2_HPO_4_ · 7H_2_O, 5.5 g/L NaH_2_PO_4_ · H_2_O, 0.75 g/L KCl, 1 mL of 1 M MgSO_4_). Cells were permeabilized by mixing them with 16 μl of chloroform and 16 μl of 2% Sarkosyl (32, 65). Permeabilized cells (50 μl) were mixed with 100 μL of Z-buffer and 50 μL of 2-mg/mL chlorophenol red–β-d-galactopyranoside (CPRG, 50 μl; Research Products International), which is considerably more sensitive than ONPG (*o*-nitrophenyl-β-d-galactopyranoside) (66). The OD_600_ was measured at the beginning of each assay. The OD_578_ was measured over time using an Infinite M200 Pro plate reader (Tecan). β-Galactosidase activity units [(micromoles of chlorophenol red formed per minute) × 10^3^/(OD_600_ × milliliters of cell suspension)] were calculated as previously described (67). Experiments were performed in technical and biological triplicates, and the means and standard deviations are shown.

### MIC assay.

To determine the MICs of various antibiotics, we diluted overnight cultures of bacteria (washed in LB) 1:1,000 in media containing 2-fold dilutions of each antibiotic. All MIC experiments were performed in round-bottom 96-well plates. Each experiment was performed in triplicate, and cells were allowed to incubate for 24 h at 37°C before we observed growth or no growth by centrifuging the plates at 1,000 rpm for 5 min and observing the presence or absence of pellets.

### Immunoblot analysis.

Cells were subcultured 1:50 and grown at 37°C to an OD_600_ of 0.6 to 0.8 in a roller drum. One-milliliter aliquots were spun down at 8,000 rpm and resuspended in 100 μL of LB with or without cefoxitin (5 μg/mL). The cells were incubated for 1 h at 37°C in a roller drum. Sample buffer was added after incubation. Samples were electrophoresed on a 15% SDS-polyacrylamide gel, and proteins were then blotted onto a nitrocellulose membrane (GE Healthcare, Amersham). Nitrocellulose was blocked with 5% bovine serum albumin (BSA), and proteins were detected with 1:10,000 anti-GFP antisera. Streptavidin IR680LT (1:10,000) was used to detect two biotin-containing proteins, PycA (HD73_4231) and AccB (HD73_4487), which serve as loading controls (68). To detect primary antibodies, the blots were incubated with 1:10,000 goat anti-rabbit IR800CW (Li-Cor) and imaged on an Azure Sapphire imager (Azure Biosystems). All immunoblots were performed at room temperature a minimum of three times, and a representative example is shown.

### Bocillin FL labeling assay.

Overnight cultures grown at 30°C were diluted 1:50 and grown to an OD of ∼1.0. The cultures were divided in 1-mL aliquots and pelleted at 8,000 rpm. The cells were washed twice in 500 μL phosphate-buffered saline (PBS) and resuspended in 50 μL PBS containing 50 μg/mL Bocillin FL for 30 min at room temperature (ThermoFisher). After incubation in Bocillin FL, all the samples were pelleted and resuspended in 200 μL sample buffer with 5% β-mercaptoethanol (βME). The samples were sonicated, heated, and electrophoresed on a 12% polyacrylamide gel. The gels were imaged on an Azure Sapphire imager (Azure Biosystems) by exciting the cells at 488 nm and detecting them at 518 nm. The Bocillin FL labeling experiment was performed in biological triplicate.

## References

[1] Staroń A, Sofia HJ, Dietrich S, Ulrich LE, Liesegang H, Mascher T. 2009. The third pillar of bacterial signal transduction: classification of the extracytoplasmic function (ECF) sigma factor protein family. Mol Microbiol 74:557–581. doi:10.1111/j.1365-2958.2009.06870.x. 19737356

[2] Casas-Pastor D, Müller RR, Jaenicke S, Brinkrolf K, Becker A, Buttner MJ, Gross CA, Mascher T, Goesmann A, Fritz G. 2021. Expansion and re-classification of the extracytoplasmic function (ECF) σ factor family. Nucleic Acids Res 49:986–1005. doi:10.1093/nar/gkaa1229. 33398323 PMC7826278

[3] Alba BM, Leeds JA, Onufryk C, Lu CZ, Gross CA. 2002. DegS and YaeL participate sequentially in the cleavage of RseA to activate the sigma(E)-dependent extracytoplasmic stress response. Genes Dev 16:2156–2168. doi:10.1101/gad.1008902. 12183369 PMC186436

[4] Schöbel S, Zellmeier S, Schumann W, Wiegert T. 2004. The *Bacillus subtilis* sigmaW anti-sigma factor RsiW is degraded by intramembrane proteolysis through YluC. Mol Microbiol 52:1091–1105. doi:10.1111/j.1365-2958.2004.04031.x. 15130127

[5] Ellermeier CD, Losick R. 2006. Evidence for a novel protease governing regulated intramembrane proteolysis and resistance to antimicrobial peptides in *Bacillus subtilis*. Genes Dev 20:1911–1922. doi:10.1101/gad.1440606. 16816000 PMC1522089

[6] Castro AN, Lewerke LT, Hastie JL, Ellermeier CD. 2018. Signal peptidase is necessary and sufficient for site 1 cleavage of RsiV in *Bacillus subtilis* in response to lysozyme. J Bacteriol 200:e00663-17. doi:10.1128/JB.00663-17. 29358498 PMC5952393

[7] Hastie JL, Williams KB, Ellermeier CD. 2013. The activity of σ^V^, an extracytoplasmic function σ factor of *Bacillus subtilis*, is controlled by regulated proteolysis of the anti-σ factor RsiV. J Bacteriol 195:3135–3144. doi:10.1128/JB.00292-13. 23687273 PMC3697642

[8] Ho TD, Nauta KM, Müh U, Ellermeier CD. 2019. Activation of the extracytoplasmic function σ factor σP by β-lactams in *Bacillus thuringiensis* requires the site-2 protease RasP. mSphere 4:e00511-19. doi:10.1128/mSphere.00511-19. 31391284 PMC6686233

[9] Campbell EA, Tupy JL, Gruber TM, Wang S, Sharp MM, Gross CA, Darst SA. 2003. Crystal structure of *Escherichia coli* sigmaE with the cytoplasmic domain of its anti-sigma RseA. Mol Cell 11:1067–1078. doi:10.1016/s1097-2765(03)00148-5. 12718891

[10] De Las Peñas A, Connolly L, Gross CA. 1997. The sigmaE-mediated response to extracytoplasmic stress in *Escherichia coli* is transduced by RseA and RseB, two negative regulators of sigmaE. Mol Microbiol 24:373–385. doi:10.1046/j.1365-2958.1997.3611718.x. 9159523

[11] Mecsas J, Rouviere PE, Erickson JW, Donohue TJ, Gross CA. 1993. The activity of sigma E, an *Escherichia coli* heat-inducible sigma-factor, is modulated by expression of outer membrane proteins. Genes Dev 7:2618–2628. doi:10.1101/gad.7.12b.2618. 8276244

[12] Walsh NP, Alba BM, Bose B, Gross CA, Sauer RT. 2003. OMP peptide signals initiate the envelope-stress response by activating DegS protease via relief of inhibition mediated by its PDZ domain. Cell 113:61–71. doi:10.1016/s0092-8674(03)00203-4. 12679035

[13] Kanehara K, Ito K, Akiyama Y. 2003. YaeL proteolysis of RseA is controlled by the PDZ domain of YaeL and a Gln-rich region of RseA. EMBO J 22:6389–6398. doi:10.1093/emboj/cdg602. 14633997 PMC291843

[14] Kim DY, Jin KS, Kwon E, Ree M, Kim KK. 2007. Crystal structure of RseB and a model of its binding mode to RseA. Proc Natl Acad Sci USA 104:8779–8784. doi:10.1073/pnas.0703117104. 17496148 PMC1885579

[15] Grigorova IL, Chaba R, Zhong HJ, Alba BM, Rhodius V, Herman C, Gross CA. 2004. Fine-tuning of the *Escherichia coli* sigmaE envelope stress response relies on multiple mechanisms to inhibit signal-independent proteolysis of the transmembrane anti-sigma factor, RseA. Genes Dev 18:2686–2697. doi:10.1101/gad.1238604. 15520285 PMC525548

[16] Kim DY. 2015. Two stress sensor proteins for the expression of sigmaE regulon: DegS and RseB. J Microbiol 53:306–310. doi:10.1007/s12275-015-5112-6. 25935301

[17] Lima S, Guo MS, Chaba R, Gross CA, Sauer RT. 2013. Dual molecular signals mediate the bacterial response to outer-membrane stress. Science 340:837–841. doi:10.1126/science.1235358. 23687042 PMC3928677

[18] Pietiäinen M, Gardemeister M, Mecklin M, Leskelä S, Sarvas M, Kontinen VP. 2005. Cationic antimicrobial peptides elicit a complex stress response in *Bacillus subtilis* that involves ECF-type sigma factors and two-component signal transduction systems. Microbiology (Reading) 151:1577–1592. doi:10.1099/mic.0.27761-0. 15870467

[19] Butcher BG, Helmann JD. 2006. Identification of *Bacillus subtilis* sigma-dependent genes that provide intrinsic resistance to antimicrobial compounds produced by Bacilli. Mol Microbiol 60:765–782. doi:10.1111/j.1365-2958.2006.05131.x. 16629676

[20] Heinrich J, Wiegert T. 2006. YpdC determines site-1 degradation in regulated intramembrane proteolysis of the RsiW anti-sigma factor of *Bacillus subtilis*. Mol Microbiol 62:566–579. doi:10.1111/j.1365-2958.2006.05391.x. 17020587

[21] Heinrich J, Hein K, Wiegert T. 2009. Two proteolytic modules are involved in regulated intramembrane proteolysis of *Bacillus subtilis* RsiW. Mol Microbiol 74:1412–1426. doi:10.1111/j.1365-2958.2009.06940.x. 19889088

[22] Zellmeier S, Schumann W, Wiegert T. 2006. Involvement of Clp protease activity in modulating the *Bacillus subtilis* sigmaW stress response. Mol Microbiol 61:1569–1582. doi:10.1111/j.1365-2958.2006.05323.x. 16899079

[23] Le Jeune A, Torelli R, Sanguinetti M, Giard J-CC, Hartke A, Auffray Y, Benachour A. 2010. The extracytoplasmic function sigma factor SigV plays a key role in the original model of lysozyme resistance and virulence of *Enterococcus faecalis*. PLoS One 5:e9658. doi:10.1371/journal.pone.0009658. 20300180 PMC2836378

[24] Ho TD, Ellermeier CD. 2011. PrsW is required for colonization, resistance to antimicrobial peptides, and expression of extracytoplasmic function σ factors in *Clostridium difficile*. Infect Immun 79:3229–3238. doi:10.1128/IAI.00019-11. 21628514 PMC3147581

[25] Ho TD, Williams KB, Chen Y, Helm RF, Popham DL, Ellermeier CD. 2014. *Clostridium difficile* extracytoplasmic function σ factor σV regulates lysozyme resistance and is necessary for pathogenesis in the hamster model of infection. Infect Immun 82:2345–2355. doi:10.1128/IAI.01483-13. 24664503 PMC4019185

[26] Ho TD, Ellermeier CD. 2019. Activation of the extracytoplasmic function σ factor σ^V^ by lysozyme. Mol Microbiol 112:410–419. doi:10.1111/mmi.14348. 31286585 PMC6703919

[27] Ho TD, Ellermeier CD. 2022. Activation of the extracytoplasmic function σ factor σ^V^ by lysozyme in *Clostridioides difficile*. Curr Opin Microbiol 65:162–166. doi:10.1016/j.mib.2021.11.008. 34894542 PMC8792214

[28] Yoshimura M, Asai K, Sadaie Y, Yoshikawa H. 2004. Interaction of *Bacillus subtilis* extracytoplasmic function (ECF) sigma factors with the N-terminal regions of their potential anti-sigma factors. Microbiology (Reading) 150:591–599. doi:10.1099/mic.0.26712-0. 14993308

[29] Zellmeier S, Hofmann C, Thomas S, Wiegert T, Schumann W. 2005. Identification of sigma(V)-dependent genes of *Bacillus subtilis*. FEMS Microbiol Lett 253:221–229. doi:10.1016/j.femsle.2005.09.056. 16274938

[30] Hastie JL, Williams KB, Sepúlveda C, Houtman JC, Forest KT, Ellermeier CD. 2014. Evidence of a bacterial receptor for lysozyme: binding of lysozyme to the anti-σ factor RsiV controls activation of the ECF σ factor σ^V^. PLoS Genet 10:e1004643. doi:10.1371/journal.pgen.1004643. 25275625 PMC4183432

[31] Hastie JL, Williams KB, Bohr LL, Houtman JC, Gakhar L, Ellermeier CD. 2016. The anti-sigma factor RsiV is a bacterial receptor for lysozyme: co-crystal structure determination and demonstration that binding of lysozyme to RsiV is required for σ^V^ activation. PLoS Genet 12:e1006287. doi:10.1371/journal.pgen.1006287. 27602573 PMC5014341

[32] Ho TD, Hastie JL, Intile PJ, Ellermeier CD. 2011. The *Bacillus subtilis* extracytoplasmic function σ factor σ^V^ is induced by lysozyme and provides resistance to lysozyme. J Bacteriol 193:6215–6222. doi:10.1128/JB.05467-11. 21856855 PMC3209206

[33] Kaus GM, Snyder LF, Müh U, Flores MJ, Popham DL, Ellermeier CD. 2020. Lysozyme resistance in *Clostridioides difficile* is dependent on two peptidoglycan deacetylases. J Bacteriol 202:e00421-20. doi:10.1128/JB.00421-20. 32868404 PMC7585060

[34] Varahan S, Iyer VS, Moore WT, Hancock LE. 2013. Eep confers lysozyme resistance to *Enterococcus faecalis* via the activation of the extracytoplasmic function sigma factor SigV. J Bacteriol 195:3125–3134. doi:10.1128/JB.00291-13. 23645601 PMC3697634

[35] Parthasarathy S, Wang X, Carr KR, Varahan S, Hancock EB, Hancock LE. 2021. SigV mediates lysozyme resistance in *Enterococcus faecalis* via RsiV and PgdA. J Bacteriol 203:e00258-21. doi:10.1128/JB.00258-21. PMC8459761 34370556

[36] Lewerke LT, Kies PJ, Müh U, Ellermeier CD. 2018. Bacterial sensing: a putative amphipathic helix in RsiV is the switch for activating σ^V^ in response to lysozyme. PLoS Genet 14:e1007527. doi:10.1371/journal.pgen.1007527. 30020925 PMC6066255

[37] Paetzel M. 2014. Structure and mechanism of *Escherichia coli* type I signal peptidase. Biochim Biophys Acta 1843:1497–1508. doi:10.1016/j.bbamcr.2013.12.003. 24333859

[38] van Roosmalen ML, Geukens N, Jongbloed JDH, Tjalsma H, Dubois J-YF, Bron S, van Dijl JM, Anné J. 2004. Type I signal peptidases of Gram-positive bacteria. Biochim Biophys Acta 1694:279–297. doi:10.1016/j.bbamcr.2004.05.006. 15546672

[39] Tjalsma H, Noback MA, Bron S, Venema G, Yamane K, Dijl JMV. 1997. *Bacillus subtilis* contains four closely related type I signal peptidases with overlapping substrate specificities. J Biol Chem 272:25983–25992. doi:10.1074/jbc.272.41.25983. 9325333

[40] Antelmann H, Tjalsma H, Voigt B, Ohlmeier S, Bron S, van Dijl JM, Hecker M. 2001. A proteomic view on genome-based signal peptide predictions. Genome Res 11:1484–1502. doi:10.1101/gr.182801. 11544192

[41] Tjalsma H, Bolhuis A, van Roosmalen ML, Wiegert T, Schumann W, Broekhuizen CP, Quax WJ, Venema G, Bron S, van Dijl JM. 1998. Functional analysis of the secretory precursor processing machinery of *Bacillus subtilis*: identification of a eubacterial homolog of archaeal and eukaryotic signal peptidases. Genes Dev 12:2318–2331. doi:10.1101/gad.12.15.2318. 9694797 PMC317044

[42] Bolhuis A, Sorokin A, Azevedo V, Ehrlich SD, Braun PG, de Jong A, Venema G, Bron S, van Dijl JM. 1996. *Bacillus subtilis* can modulate its capacity and specificity for protein secretion through temporally controlled expression of the sipS gene for signal peptidase I. Mol Microbiol 22:605–618. doi:10.1046/j.1365-2958.1996.d01-4676.x. 8951809

[43] Ross CL, Thomason KS, Koehler TM. 2009. An extracytoplasmic function sigma factor controls beta-lactamase gene expression in *Bacillus anthracis* and other *Bacillus cereus* group species. J Bacteriol 191:6683–6693. doi:10.1128/JB.00691-09. 19717606 PMC2795285

[44] Nauta KM, Ho TD, Ellermeier CD. 2021. The penicillin-binding protein PbpP is a sensor of β-lactams and is required for activation of the extracytoplasmic function σ factor σ^P^ in *Bacillus thuringiensis*. mBio 12:e00179-21. doi:10.1128/mBio.00179-21. 33758089 PMC8092216

[45] Chen Y, Succi J, Tenover FC, Koehler TM. 2003. β-Lactamase genes of the penicillin-susceptible *Bacillus anthracis* Sterne strain. J Bacteriol 185:823–830. doi:10.1128/JB.185.3.823-830.2003. 12533457 PMC142833

[46] Chen Y, Tenover FC, Koehler TM. 2004. Beta-lactamase gene expression in a penicillin-resistant *Bacillus anthracis* strain. Antimicrob Agents Chemother 48:4873–4877. doi:10.1128/AAC.48.12.4873-4877.2004. 15561870 PMC529205

[47] Gargis AS, Lascols C, McLaughlin HP, Conley AB, Hoffmaster AR, Sue D. 2019. Genome sequences of penicillin-resistant *Bacillus anthracis* strains. Microbiol Resour Announc 8:e01122-18. doi:10.1128/MRA.01122-18. 30643874 PMC6328647

[48] Gargis AS, McLaughlin HP, Conley AB, Lascols C, Michel PA, Gee JE, Marston CK, Kolton CB, Rodriguez-R LM, Hoffmaster AR, Weigel LM, Sue D. 2018. Analysis of whole-genome sequences for the prediction of penicillin resistance and β-lactamase activity in *Bacillus anthracis*. mSystems 3:e00154-18. doi:10.1128/mSystems.00154-18. PMC6290263 30574557

[49] Neef J, Bongiorni C, Goosens VJ, Schmidt B, van Dijl JM. 2017. Intramembrane protease RasP boosts protein production in *Bacillus*. Microb Cell Fact 16:57. doi:10.1186/s12934-017-0673-1. 28376795 PMC5381017

[50] Almagro Armenteros JJ, Tsirigos KD, Sønderby CK, Petersen TN, Winther O, Brunak S, von Heijne G, Nielsen H. 2019. SignalP 5.0 improves signal peptide predictions using deep neural networks. Nat Biotechnol 37:420–423. doi:10.1038/s41587-019-0036-z. 30778233

[51] Altschul SF, Madden TL, Schäffer AA, Zhang J, Zhang Z, Miller W, Lipman DJ. 1997. Gapped BLAST and PSI-BLAST: a new generation of protein database search programs. Nucleic Acids Res 25:3389–3402. doi:10.1093/nar/25.17.3389. 9254694 PMC146917

[52] Burdon KL. 1956. Useful criteria for the identification of *Bacillus anthracis* and related species. J Bacteriol 71:25–42. doi:10.1128/jb.71.1.25-42.1956. 13286227 PMC357732

[53] Brown ER, Moody MD, Treece EL, Smith CW. 1958. Differential diagnosis of *Bacillus cereus, Bacillus anthracis*, and *Bacillus cereus* var. mycoides. J Bacteriol 75:499–509. doi:10.1128/jb.75.5.499-509.1958. 13538915 PMC290100

[54] Sineva E, Savkina M, Ades SE. 2017. Themes and variations in gene regulation by extracytoplasmic function (ECF) sigma factors. Curr Opin Microbiol 36:128–137. doi:10.1016/j.mib.2017.05.004. 28575802 PMC5534382

[55] Mascher T. 2013. Signaling diversity and evolution of extracytoplasmic function (ECF) σ factors. Curr Opin Microbiol 16:148–155. doi:10.1016/j.mib.2013.02.001. 23466210

[56] Tuteja R. 2005. Type I signal peptidase: an overview. Arch Biochem Biophys 441:107–111. doi:10.1016/j.abb.2005.07.013. 16126156

[57] Wilcks A, Jayaswal N, Lereclus D, Andrupl L. 1998. Characterization of plasmid pAW63, a second self-transmissible plasmid in Bacillus thuringiensis subsp. kurstaki HD73. Microbiology (Reading) 144:1263–1270. doi:10.1099/00221287-144-5-1263. 9611801

[58] Youngman P, Perkins JB, Losick R. 1984. Construction of a cloning site near one end of Tn917 into which foreign DNA may be inserted without affecting transposition in *Bacillus subtilis* or expression of the transposon-borne erm gene. Plasmid 12:1–9. doi:10.1016/0147-619x(84)90061-1. 6093169

[59] Gibson DG, Young L, Chuang R, Venter JC, Hutchison CA, Smith HO. 2009. Enzymatic assembly of DNA molecules up to several hundred kilobases. Nat Methods 6:343–345. doi:10.1038/nmeth.1318. 19363495

[60] Arnaud M, Chastanet A, Débarbouillé M. 2004. New vector for efficient allelic replacement in naturally nontransformable, low-GC-content, gram-positive bacteria. Appl Environ Microbiol 70:6887–6891. doi:10.1128/AEM.70.11.6887-6891.2004. 15528558 PMC525206

[61] Brophy JAN, Triassi AJ, Adams BL, Renberg RL, Stratis-Cullum DN, Grossman AD, Voigt CA. 2018. Engineered integrative and conjugative elements for efficient and inducible DNA transfer to undomesticated bacteria. Nat Microbiol 3:1043–1053. doi:10.1038/s41564-018-0216-5. 30127494

[62] Bott KF, Wilson GA. 1967. Development of competence in the *Bacillus subtilis* transformation system. J Bacteriol 94:562–570. doi:10.1128/jb.94.3.562-570.1967. 4962301 PMC251923

[63] Bone EJ, Ellar DJ. 1989. Transformation of *Bacillus thuringiensis* by electroporation. FEMS Microbiol Lett 58:171–177. doi:10.1111/j.1574-6968.1989.tb03039.x. 2545517

[64] Lereclus D, Arantès O, Chaufaux J, Lecadet M. 1989. Transformation and expression of a cloned delta-endotoxin gene in *Bacillus thuringiensis*. FEMS Microbiol Lett 60:211–217. doi:10.1111/j.1574-6968.1989.tb03448.x. 2550317

[65] Griffith KL, Wolf RE. 2002. Measuring beta-galactosidase activity in bacteria: cell growth, permeabilization, and enzyme assays in 96-well arrays. Biochem Biophys Res Commun 290:397–402. doi:10.1006/bbrc.2001.6152. 11779182

[66] Eustice DC, Feldman PA, Colberg-Poley AM, Buckery RM, Neubauer RH. 1991. A sensitive method for the detection of beta-galactosidase in transfected mammalian cells. Biotechniques 11:739–740, 742–743. 1809326

[67] Slauch JM, Silhavy TJ. 1991. *cis*-acting *ompF* mutations that result in OmpR-dependent constitutive expression. J Bacteriol 173:4039–4048. doi:10.1128/jb.173.13.4039-4048.1991. 1648075 PMC208052

[68] Marini P, Li SJ, Gardiol D, Cronan JE, De Mendoza D. 1995. The genes encoding the biotin carboxyl carrier protein and biotin carboxylase subunits of *Bacillus subtilis* acetyl coenzyme A carboxylase, the first enzyme of fatty acid synthesis. J Bacteriol 177:7003–7006. doi:10.1128/jb.177.23.7003-7006.1995. 7592499 PMC177574

[69] Karp PD, Billington R, Caspi R, Fulcher CA, Latendresse M, Kothari A, Keseler IM, Krummenacker M, Midford PE, Ong Q, Ong WK, Paley SM, Subhraveti P. 2017. The BioCyc collection of microbial genomes and metabolic pathways. Brief Bioinform 20:1085–1093. doi:10.1093/bib/bbx085. PMC6781571 29447345

[70] Malone CL, Boles BR, Lauderdale KJ, Thoendel M, Kavanaugh JS, Horswill AR. 2009. Fluorescent reporters for *Staphylococcus aureus*. J Microbiol Methods 77:251–260. doi:10.1016/j.mimet.2009.02.011. 19264102 PMC2693297

[71] Guérout-Fleury AM, Frandsen N, Stragier P. 1996. Plasmids for ectopic integration in *Bacillus subtilis*. Gene 180:57–61. doi:10.1016/s0378-1119(96)00404-0. 8973347

