# Supplementary figures and images for: Signal Peptidase-Mediated Cleavage of the Anti-σ Factor RsiP at Site 1 Controls σP Activation and β-Lactam Resistance in Bacillus thuringiensis
Source: mBio. 2022 Feb 15;13(1):e03707-21. doi: 10.1128/mbio.03707-21 (PMC8844934; doi:10.1128/mbio.03707-21)

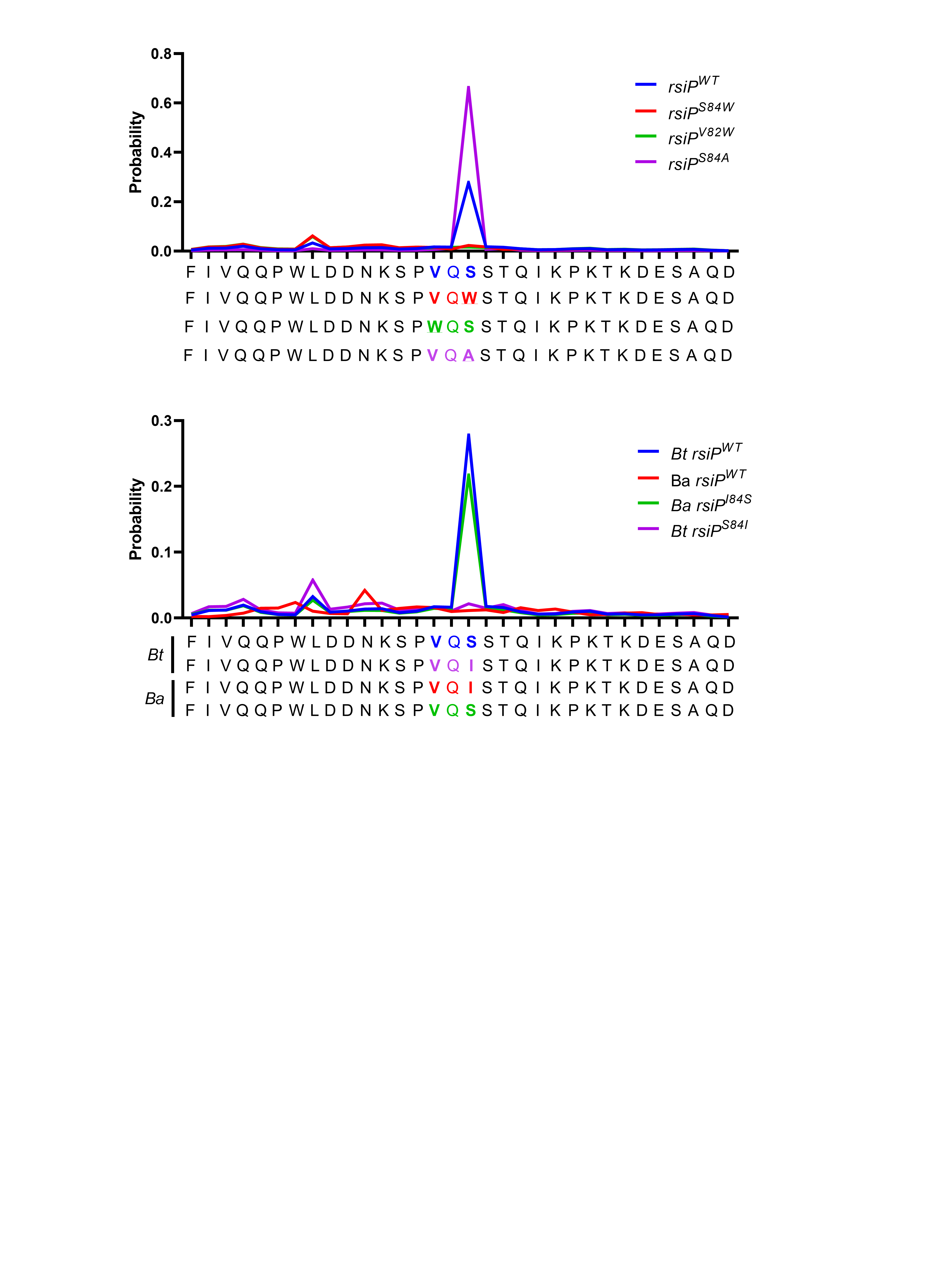

Supplement: FIG S1 [file mbio.03707-21-sf001.tif]

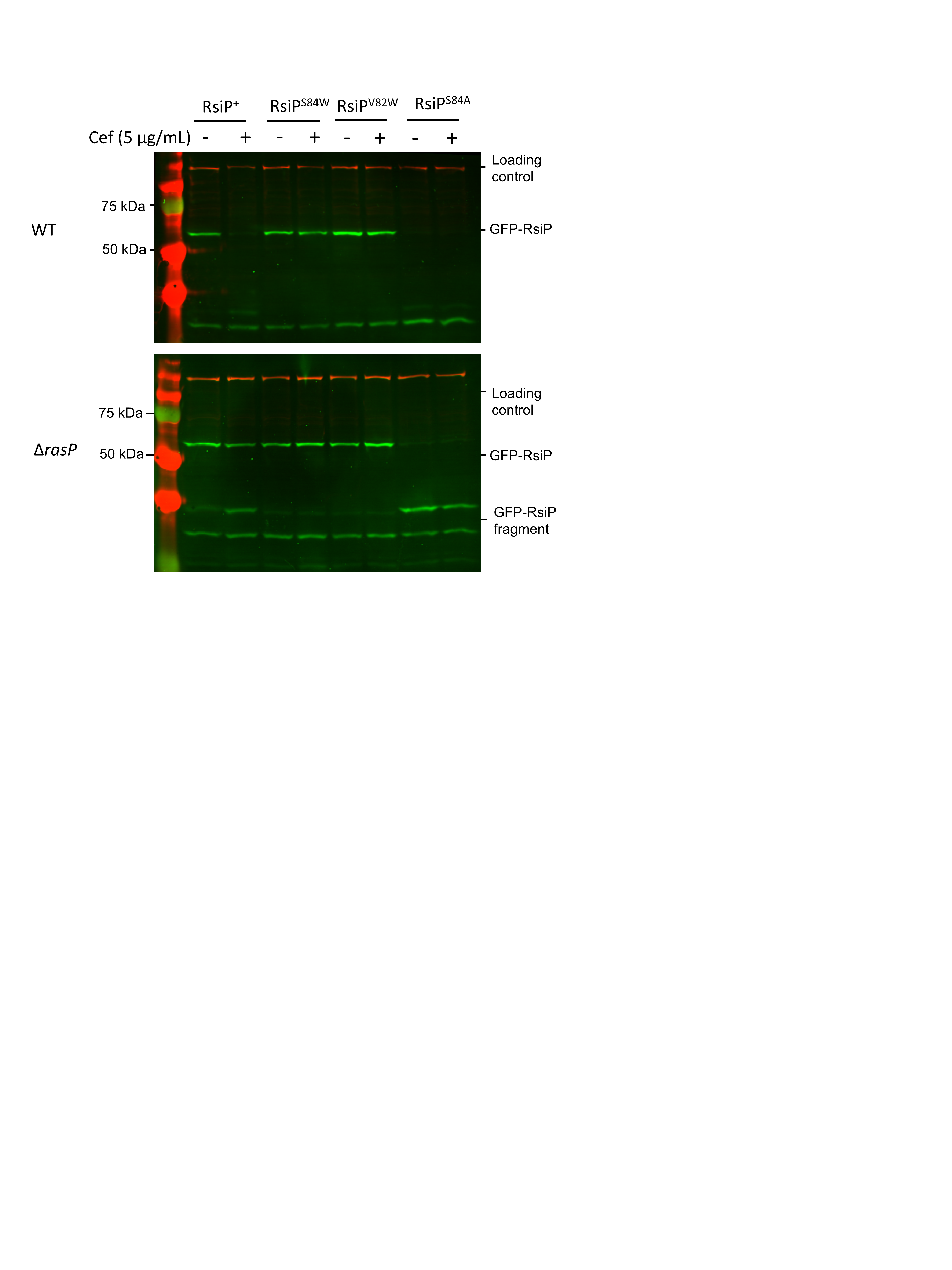

Supplement: FIG S2 [file mbio.03707-21-sf002.tif]

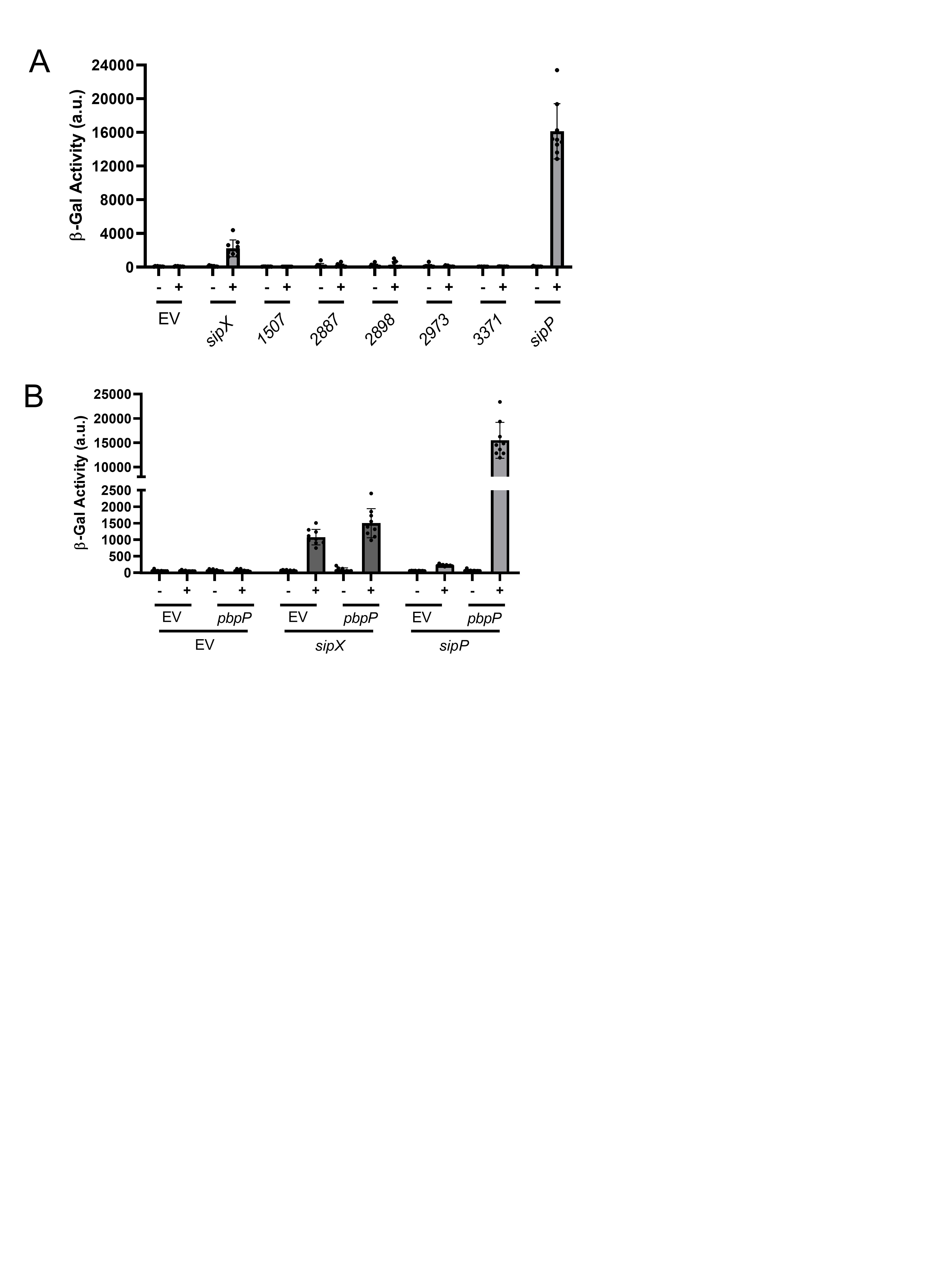

Supplement: FIG S3 [file mbio.03707-21-sf003.tif]

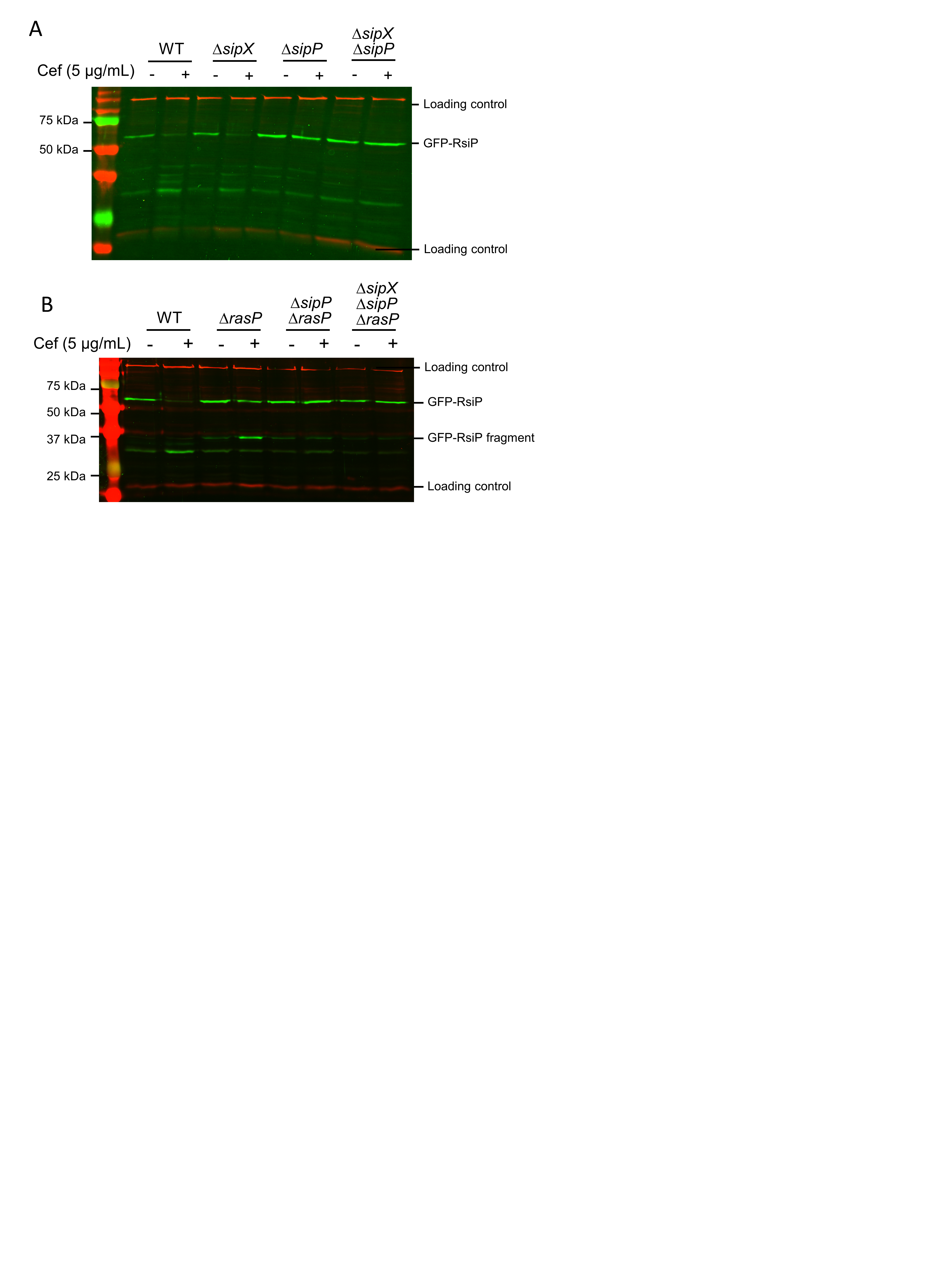

Supplement: FIG S4 [file mbio.03707-21-sf004.tif]

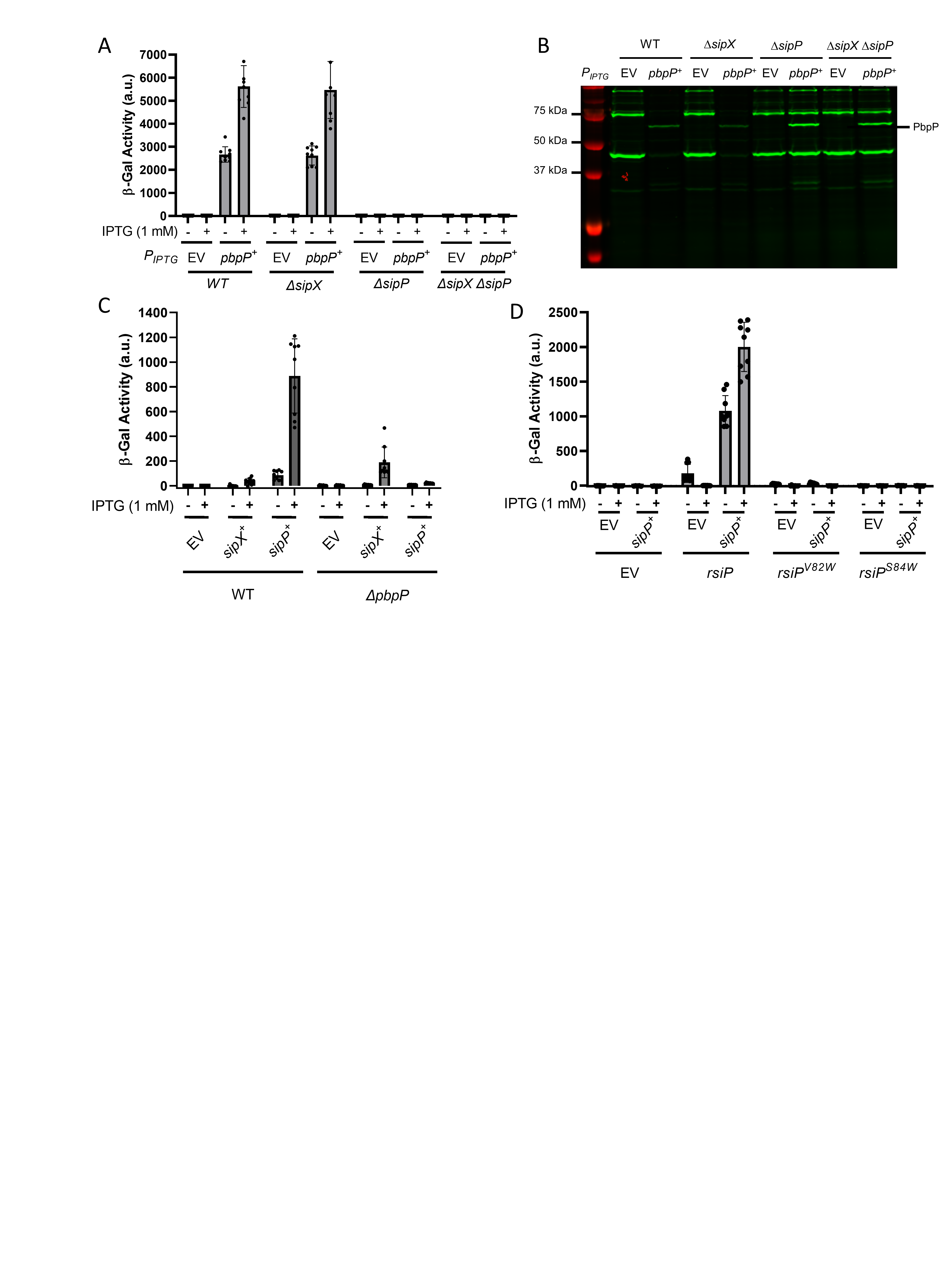

Supplement: FIG S5 [file mbio.03707-21-sf005.tif]

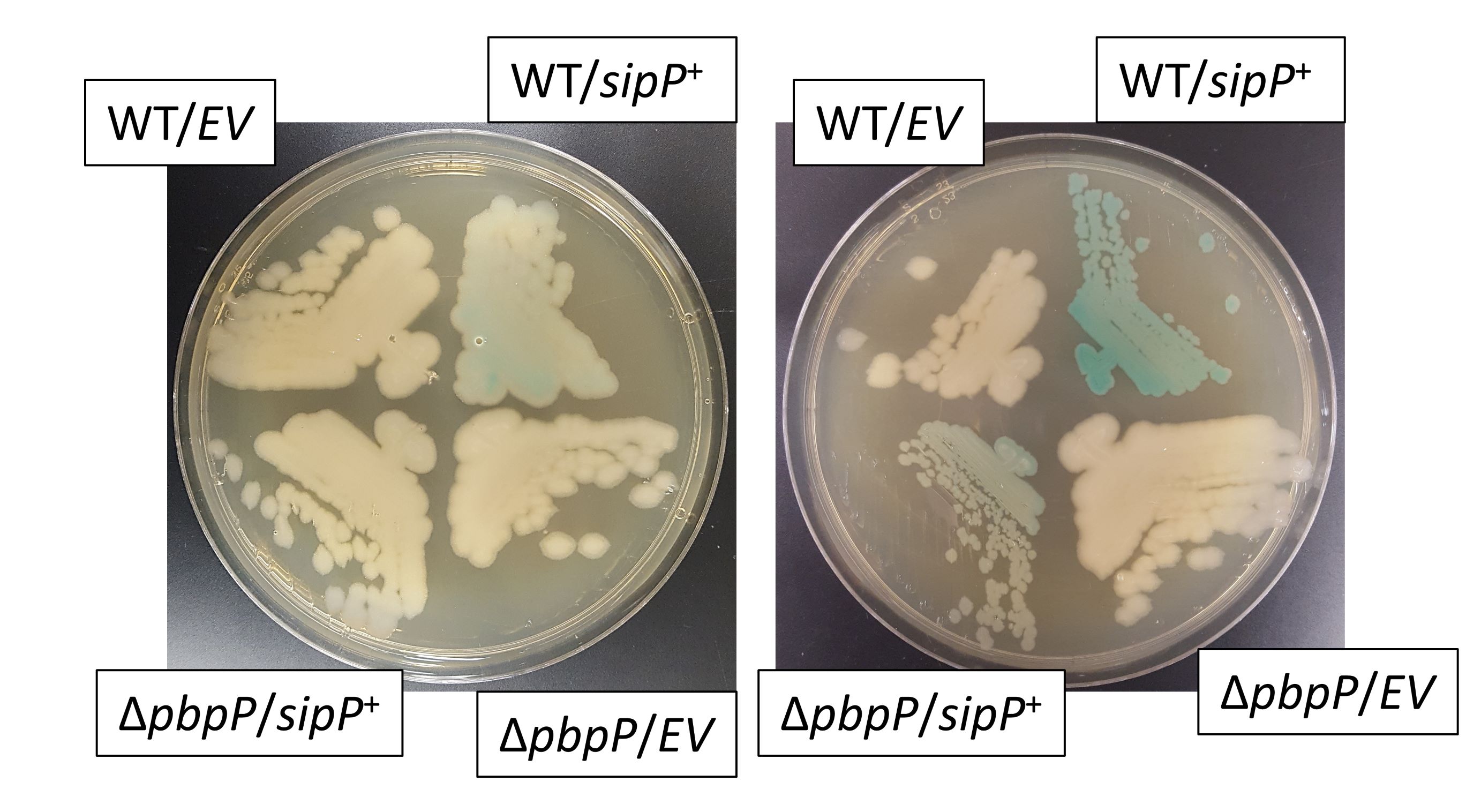

Supplement: FIG S6 [file mbio.03707-21-sf006.jpg]

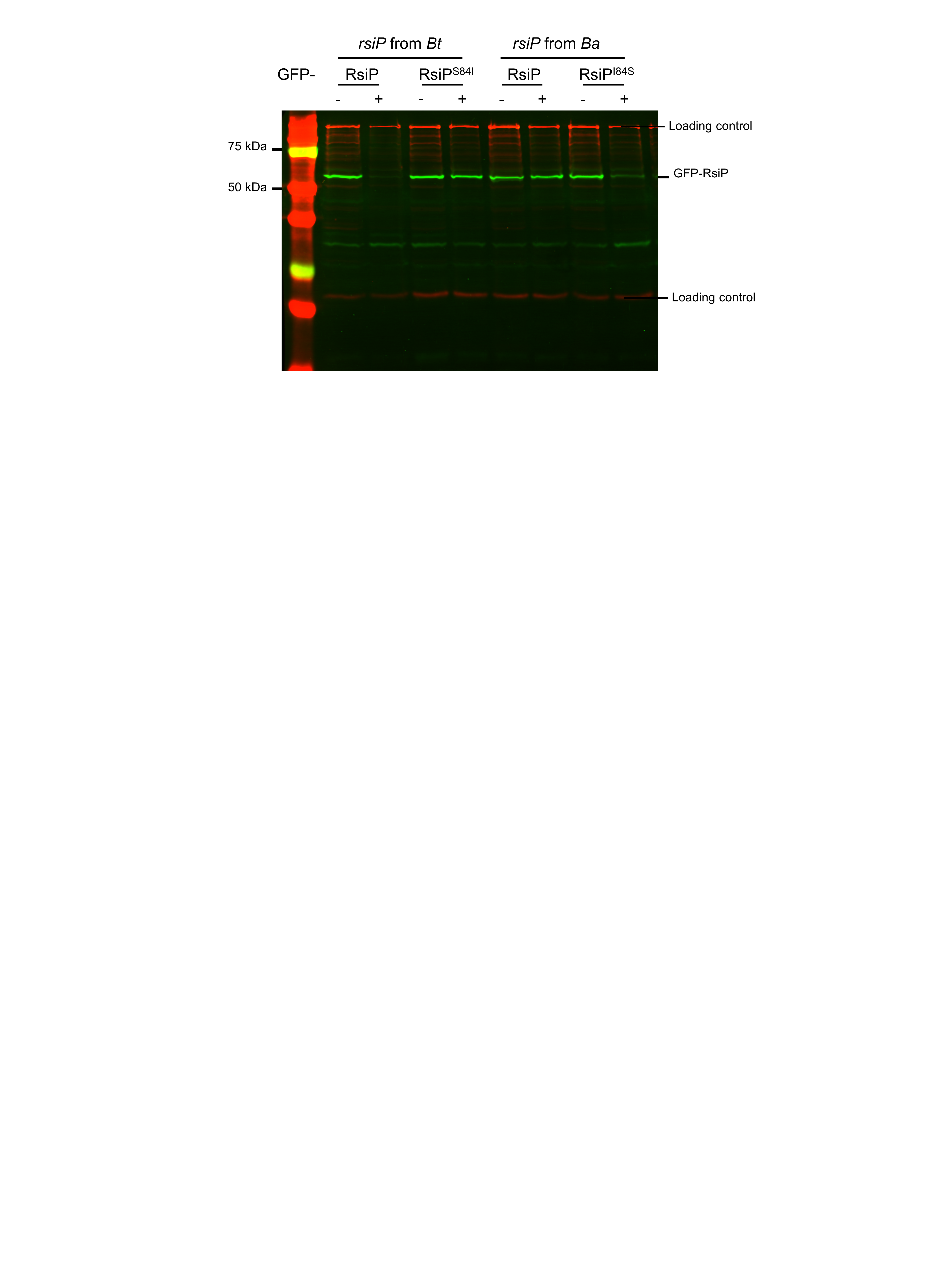

Supplement: FIG S7 [file mbio.03707-21-sf007.tif]

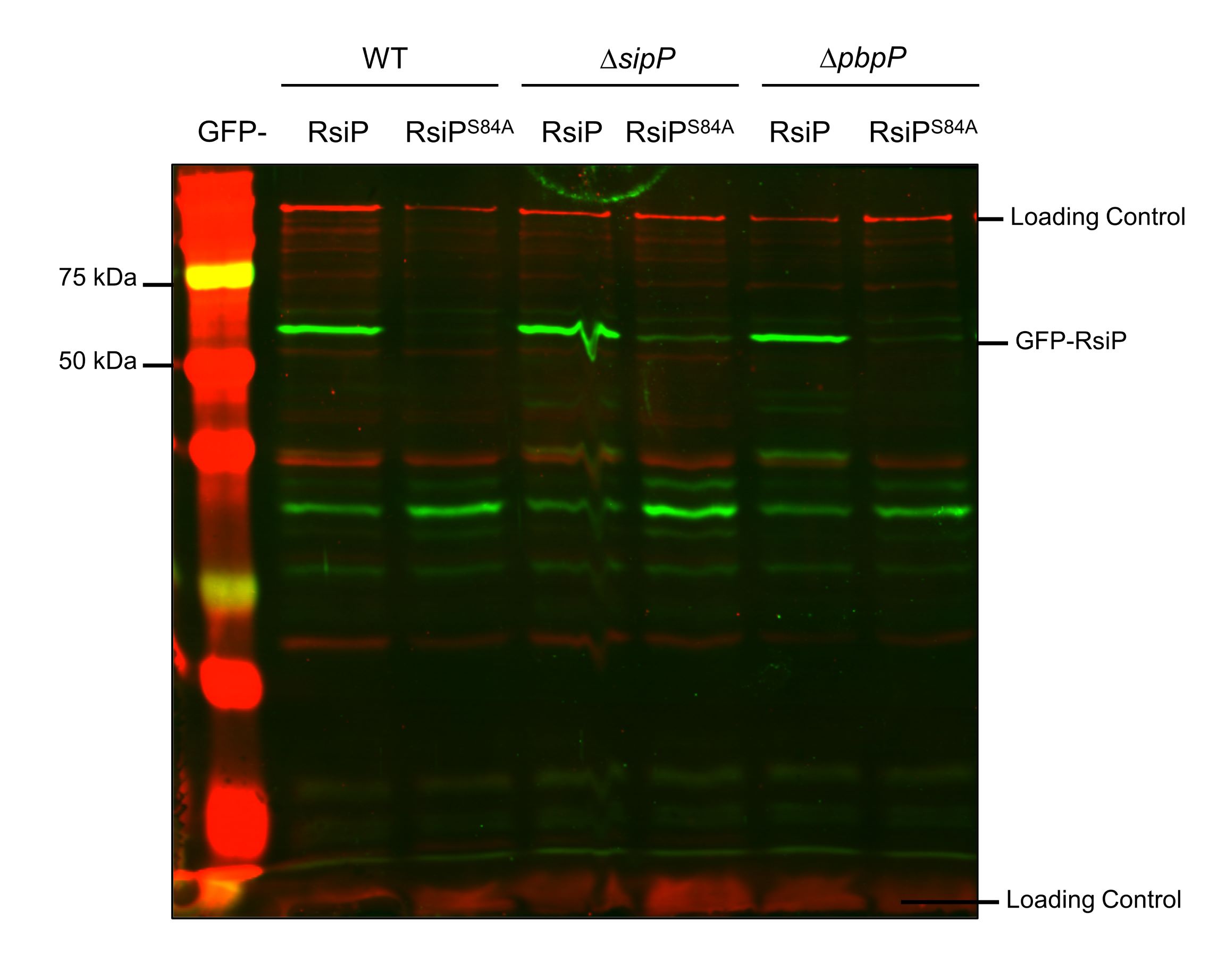

Supplement: FIG S8 [file mbio.03707-21-sf008.jpg]
