# Supplementary material for: Signal Peptidase-Mediated Cleavage of the Anti-σ Factor RsiP at Site 1 Controls σP Activation and β-Lactam Resistance in Bacillus thuringiensis
Source: mBio. 2022 Feb 15;13(1):e03707-21. doi: 10.1128/mbio.03707-21 (PMC8844934; doi:10.1128/mbio.03707-21)
Supplement: TABLE S1 [file mbio.03707-21-st001.pdf]

TABLE S1. Plasmids used in this study

Table S1. Plasmids

Plasmid	Relevant features	Parent vector	Restriction enzymes to digest parent vector	PCR primers	PCR template	Reference
pMAD	ori-pE194ts , *amp, erm*					61
pAH9	ori-pE194 P_{sarA} *-mcherry* , *amp, erm*					70
pDR160	*amyE* ::P_{xyl} *amp, spec*					David Rudner
pDG1663	*thrC::lacZ erm, amp*					71
pDR111	*amyE* ::P_{IPTG} *amp, spec*					David Rudner
pCE695	*amyE* ::P_{IPTG}-*gfp-rsiP* *amp, spec*					44
pJAB980	ICE::P_{IPTG} *-gfp* *amp, cat*					52
pCE697	ICEBs1::P_{IPTG} *amp, cat*					44
pCE698	ICEBs1::P_{IPTG}-*gfp-rsiP* *amp, cat*					44
pCE707	ICE::P_{IPTG} *-pbpP*$^{+}$ *amp, cat*					44
pTHE960	ori-pE194, P_{sigP} *-sigP*$^{+}$ *rsiP*$^{+}$ *amp, erm*					8
pCE868	ICEBs1::P_{IPTG}-*gfp-rsiP*^{S84W} *amp, cat*	pJAB980	SalI, NheI	4515-5079; 5078-4516	pCE698	This study
pCE869	ICEBs1::P_{IPTG}-*gfp-rsiP*^{V82W} *amp, cat*	pJAB980	SalI, NheI	4515-5177; 5176-4516	pCE698	This study
pCE870	ICEBs1::P_{IPTG}-*gfp-rsiP*^{S84A} *amp, cat*	pJAB980	SalI, NheI	4515-5179; 5178-4516	pCE698	This study
pCE832	ori-pE194, P_{sigP} *-sigP*$^{+}$ *rsiP*^{S84W} *amp, erm*	pAH9	HindIII, EcoRI	3774-5079; 5078-3775	pTHE960	This study
pCE846	ori-pE194, P_{sigP} *-sigP*$^{+}$ *rsiP*^{V82W} *amp, erm*	pAH9	HindIII, EcoRI	3774-5177; 5176-3775	pTHE960	This study
pCE851	ori-pE194, P_{sigP} *-sigP*$^{+}$ *rsiP*^{S84A} *amp, erm*	pAH9	HindIII, EcoRI	3774-5179; 5178-3775	pTHE960	This study
pCE834	ICE::P_{IPTG} *-sipX*$^{+}$ *amp, cat*	pJAB980	SalI, NheI	5154-5155	AW43	This study
pCE835	ICE::P_{IPTG} *-bt1507*$^{+}$ *amp, cat*	pJAB980	SalI, NheI	5156-5157	AW43	This study
pCE833	ICE::P_{IPTG} *-bt2887*$^{+}$ *amp, cat*	pJAB980	SalI, NheI	5150-5151	AW43	This study
pCE847	ICE::P_{IPTG} *-bt2898*$^{+}$ *amp, cat*	pJAB980	SalI, NheI	5158-5159	AW43	This study
pCE836	ICE::P_{IPTG} *-bt2973*$^{+}$ *amp, cat*	pJAB980	SalI, NheI	5160-5161	AW43	This study
pCE838	ICE::P_{IPTG} *-bt3371*$^{+}$ *amp, cat*	pJAB980	SalI, NheI	5164-5165	AW43	This study
pCE840	ICE::P_{IPTG} *-sipP*$^{+}$ *(bt4122)* *amp, cat*	pJAB980	SalI, NheI	5168-5169	AW43	This study
pCE897	ICEBs1::P_{IPTG}-*gfp-rsiP*^{S84I} *amp, cat*	pJAB980	SalI, NheI	4515-5324; 5323-4516	pCE698	This study
pCE905	ICEBs1::P_{IPTG}-*gfp-rsiP(Ba sterne)* *amp, cat*	pJAB980	SalI, NheI	4515-5318; 5319-5320	pCE698; *B. anthracis* Sterne	This study
pCE906	ICEBs1::P_{IPTG}-*gfp-rsiP*^{I84S} *(Ba sterne)* *amp, cat*	pJAB980	SalI, NheI	4515-5323; 5322-5320	pCE905	This study
pCE852	Δ*sipX* ori-pE194ts , *amp, erm*	pMAD	EcoRI, BglII	5196-5197; 5198-5199	AW43	This study
pCE853	Δ*sipP* ori-pE194ts , *amp, erm*	pMAD	EcoRI, BglII	5200-5201; 5202-5203	AW43	This study
pCE795	*amyE* ::P_{xyl} *-pbpP*$^{+}$ *amp, spec*	pDR160	NheI, BamHI	4996-4997	AW43	This study
pCE811	*thrC::P* $_{sigP}$ *-sigP*$^{+}$ *rsiP*$^{+}$ *-lacZ erm, amp*	pDG1663	EcoRI, BamHI	5055-5056	AW43	This study
