# Supplementary material for: Signal Peptidase-Mediated Cleavage of the Anti-σ Factor RsiP at Site 1 Controls σP Activation and β-Lactam Resistance in Bacillus thuringiensis
Source: mBio. 2022 Feb 15;13(1):e03707-21. doi: 10.1128/mbio.03707-21 (PMC8844934; doi:10.1128/mbio.03707-21)
Supplement: TABLE S2 [file mbio.03707-21-st002.pdf]

TABLE S2. Primers used in this study

Table S2. Primers

Oligo	Sequence	Relevant features
CDEP4515	taacaattaagcttagtcgaagatctgagctcctgcagta	Clone GFP-RsiP onto pJAB980 digested w/ SalI and NheI
CDEP4516	attagcttgcatgcggctagttaccatatagaatctatatttattattttcca	Clone GFP-RsiP onto pJAB980 digested w/ SalI and NheI
CDEP3774	acgacggccagtgccaagctaattatatggtgaattttttgactaaattt	clone PsigP-sigPrsiP onto pAH9 w/ HindIII EcoRI
CDEP3775	tatgaccatgattacgaattaaccttcctcttttaccatataga	clone PsigP-sigPrsiP onto pAH9 w/ HindIII EcoRI
CDEP5150	taacaattaagcttagtcga taaggagg gagaaacgatgaaggaaaatacgaagaa	Clone BT_2887 into pCE697 digested w/ SalI and NheI
CDEP5151	attagcttgcatgcggctaggcaaataaaaaagcatggaagaa	Clone BT_2887 into pCE697 digested w/ SalI and NheI
CDEP5154	taacaattaagcttagtcgataaggaggttggtgaaatgatgcaaaagaaaaaacg	Clone sipX into pCE697 digested w/ SalI and NheI
CDEP5155	attagcttgcatgcggctaggcaaactaaaatttgcttggttt	Clone sipX into pCE697 digested w/ SalI and NheI
CDEP5156	taacaattaagcttagtcgataaggagggagagacgatgaaattaatatggaagat	Clone BT_1507 into pCE697 digested w/ SalI and NheI
CDEP5157	attagcttgcatgcggctagcggaaagtataaccaaacgaa	Clone BT_1507 into pCE697 digested w/ SalI and NheI
CDEP5158	taacaattaagcttagtcgataaggagggggaatcgatgcagccgactttatatga	Clone BT_2898 into pCE697 digested w/ SalI and NheI
CDEP5159	attagcttgcatgcggctagggggttttgctcctctccca	Clone BT_2898 into pCE697 digested w/ SalI and NheI
CDEP5160	taacaattaagcttagtcgataaggaggtatatgcgatatggagtttggaaaaaga	Clone BT_2973 into pCE697 digested w/ SalI and NheI
CDEP5161	attagcttgcatgcggctagcccatcaataatcttctctaagaat	Clone BT_2973 into pCE697 digested w/ SalI and NheI
CDEP5164	taacaattaagcttagtcgataaggaggaggtgtatatgaagctgtttattctaaa	Clone BT_3371 into pCE697 digested w/ SalI and NheI
CDEP5165	attagcttgcatgcggctaggctattaatatatgattgattttataaaacgt	Clone BT_3371 into pCE697 digested w/ SalI and NheI
CDEP5168	taacaattaagcttagtcgataaggaggggaaatgcatgaaaaaagagaagagttc	Clone sipP into pCE697 digested w/ SalI and NheI
CDEP5169	attagcttgcatgcggctaggccctggaaaccattgaatt	Clone sipP into pCE697 digested w/ SalI and NheI
CDEP5078	gataataaatcgccggtacaa tgg agtactcaaattaagcctaaaac	rsiP S84W
CDEP5079	gttttaggcttaatttgagtactccattgtaccggcgatttattatc	rsiP S84W
CDEP5176	gatgataataaatcgccg tgg caaagtagtactcaaattaag	rsiP V82W
CDEP5177	cttaatttgagtactactttgccacggcgatttattatcatc	rsiP V82W
CDEP5178	gataataaatcgccggtacaa gca agtactcaaattaagcctaaaac	rsiP S84A
CDEP5179	gttttaggcttaatttgagtacttgcttgtaccggcgatttattatc	rsiP S84A
CDEP5196	acacattaactagacagatcaatggagcaagaaaaacaagt	Clone ∆sipX onto pMAD digested w/ BglII and EcoRI
CDEP5197	gcaaactaaaatttgcttggttcaccaactcatcatcttttt	Clone ∆sipX onto pMAD digested w/ BglII and EcoRI
CDEP5198	aaagatgatgagttggtgaaccaagcaaattttagtttgct	Clone ∆sipX onto pMAD digested w/ BglII and EcoRI
CDEP5199	ctgcagaagcttctagaattggtttacacaaaagacaattgc	Clone ∆sipX onto pMAD digested w/ BglII and EcoRI
CDEP5200	acacattaactagacagatccttcgtatgtgggcattatg	Clone ∆sipP onto pMAD digested w/ BglII and EcoRI
CDEP5201	gttgccaccttctttttcatgcatttcccctccacggtta	Clone ∆sipP onto pMAD digested w/ BglII and EcoRI
CDEP5202	taaccgtggaggggaaatgcatgaaaaagaaggtggcaac	Clone ∆sipP onto pMAD digested w/ BglII and EcoRI
CDEP5203	ctgcagaagcttctagaattcataatttctttcaatacattttctgc	Clone ∆sipP onto pMAD digested w/ BglII and EcoRI
CDEP4996	ttaattaagtcgacagctagtatgaaataggctacataaggga	Clone PbpP into pDR160 digested w/ NheI and BamHI
CDEP4997	agcgaccggcgctcaggatctcactttaatgaaccttcctct	Clone PbpP into pDR160 digested w/ NheI and BamHI
CDEP5055	aactttagggttatcgaattccaggtgaaaaagtaaaacatg	Clone PsigP-sigPrsiP onto pDG1663 digested w/ EcoRI and BamHI
CDEP5056	gtatcaacaagctggggatcgtgaaccttcctcttttacca	Clone PsigP-sigPrsiP onto pDG1663 digested w/ EcoRI and BamHI
CDEP5323	atcgccggtacaaattagtactcaaattaa	rsiP S84I
CDEP5324	ttaatttgagtactaatttgtaccggcgat	rsiP S84I
CDEP5318	aggatttttttattgttcatcttatataattcatccattccgtgt	Clone GFP-RsiP Ba sterne into pCE697 digest with SalI and NheI
CDEP5319	cggaatggatgaattatataagatgaacaataaaaaaaatcctgact	Clone GFP-RsiP Ba sterne into pCE697 digest with SalI and NheI
CDEP5320	attagcttgcatgcggctag ttaccaaatagaatctatatttattattttcca	Clone GFP-RsiP Ba sterne into pCE697 digest with SalI and NheI
CDEP5321	atcaccggtgcaaagtagcacacaaattaa	rsiP Ba sterne I84S
CDEP5322	ttaatttgtgtgctactttgcaccggtgat	rsiP Ba sterne I84S
